# Non-Hormonal Strategies in Endometriosis: Targets with Future Clinical Potential

**DOI:** 10.3390/jcm14145091

**Published:** 2025-07-17

**Authors:** Maria E. Ramos-Nino

**Affiliations:** Department of Microbiology, Immunology, and Pharmacology, St. George’s University School of Medicine, St. George P.O. Box 7, Grenada; mramosni@sgu.edu

**Keywords:** endometriosis, non-hormonal, therapy, chronic inflammatory disease

## Abstract

Endometriosis is a chronic gynecological pathology marked by the aberrant proliferation of tissue analogous to the endometrial lining outside the uterine cavity. This disorder frequently engenders persistent pelvic discomfort, infertility, and an extensive array of additional manifestations, including menorrhagia, dyspareunia, and gastrointestinal anomalies. Affecting an estimated 10% of women within the reproductive age demographic globally, endometriosis continues to present as a multifaceted and formidable challenge. The precise etiology remains elusive, leading to extended diagnostic intervals and personalized, often inadequate, therapeutic approaches. The intrinsic heterogeneity of endometriosis, evident in its varied phenotypes and clinical manifestations, further complicates both precise diagnosis and efficacious treatment. Conventional management hinges on hormonal interventions, which may not be appropriate for women desiring conception or for those experiencing substantial adverse effects. While surgical procedures are accessible, they do not provide a conclusive resolution, and the probability of recurrence remains high. Progress in diagnostic methodologies, such as non-invasive biomarker analyses, combined with an expanding understanding of the molecular and immunological frameworks that underpin the condition, presents promising prospects for the development of more targeted and individualized non-hormonal treatment modalities in the near future.

## 1. Introduction

Endometriosis is a gynecological condition that affects the quality of life of women worldwide. Ectopic growth of endometrial-like tissue outside the uterus leads to persistent pain and infertility, along with other severe symptoms [1], pain being one of the most prominent and disruptive manifestations [2]. Endometrial-like tissue can occasionally develop in unusual locations outside the pelvic cavity, including organs such as the liver, lungs, and even the brain [3,4]. Despite their frequent occurrence, the biological mechanisms of this disease remain unclear, which complicates the development of effective treatments. Hormonal treatment options are common in endometriosis management but inappropriate for patients who wish to become pregnant or those who experience negative treatment effects. The search for non-hormonal treatments that target the basic biological processes of endometriosis is gaining popularity. This review summarizes the newest scientific advancements in non-hormonal medication approaches for treating endometriosis.

Endometriosis is recognized as a heterogeneous disorder categorized into three distinct phenotypes: endometriosis presents itself primarily as superficial peritoneal endometriosis (SPE), ovarian endometriomas (OMA), and deep infiltrating endometriosis (DIE) [5]. The diversity of anatomical sites and clinical severity in different phenotypes complicates their diagnosis and treatment strategies [6]. The Revised American Society for Reproductive Medicine (rASRM) classification system defines stages of endometriosis ranging from Stage I for minimal involvement to Stage IV, which denotes severe involvement based on lesion extent and severity [7].

It has been estimated on a global scale that approximately 10% of women within their reproductive age are diagnosed with endometriosis; however, this figure escalates to between 35% and 50% among populations experiencing chronic pelvic pain or infertility [8]. Despite its major effects, the precise prevalence of endometriosis remains unclear due to long diagnostic delays ranging from 7 to 10 years and inconsistent results from different study types [9]. The prevalence estimates of endometriosis exhibit considerable variability contingent upon the specific type of data utilized in research investigations. The prevalence of endometriosis stands at around 1%, according to health insurance data; clinical investigations reveal prevalence rates around 6.8%, and population-based assessments identify figures near 6.6%. Patients selected based on their symptoms demonstrate dramatically higher prevalence rates, which can go up to 21%. The discrepancies in prevalence data highlight systematic diagnostic failures of endometriosis [10]. The widespread underdiagnosis of endometriosis on a global scale, which is especially evident in low- and middle-income nations as a result of inadequate specialized surgical capacity in gynecology, and the common misattribution of pelvic pain to non-gynecological conditions consequently delay precise diagnosis [10]. Emerging technological solutions have the potential to decrease this diagnostic inequality. The development of non-invasive techniques like menstrual blood biomarker assays stands as an effective method for early detection of diseases [9]. Research on assisted reproductive technology (ART) results confirms endometriosis as a common condition in infertile women but shows no uniform reduction in reproductive success rates [11].

This narrative review seeks to integrate the prevailing knowledge regarding non-hormonal methodologies for the management of endometriosis. Non-hormonal interventions pertain to strategies that do not alter or inhibit the physiological hormonal systems of the body, especially estrogen, which plays a pivotal role in the advancement of endometriosis. Rather, these approaches concentrate on mitigating symptoms such as pain and inflammation or addressing the pathophysiological processes of the disease while circumventing the adverse effects typically associated with hormonal therapies. The pertinent literature was discerned through comprehensive searches of databases such as PubMed, Google Scholar, and Embase, employing keywords including “drug”, “therapy”, “endometriosis”, and “non-hormonal”. Only peer-reviewed publications in the English language were evaluated, and studies were chosen based on their pertinence to the objectives of this review. No formalized assessment of quality was undertaken. The results are organized thematically to underscore significant concepts, emerging trends, and deficiencies in the current body of knowledge.

## 2. Factors Influencing Endometriosis

Endometriosis functions as a complex medical condition whose development depends on multiple factors that can increase or decrease a person’s likelihood of contracting the disease. Key determinants include the following:

a. Family History

Endometriosis is extensively acknowledged as a complex, multifactorial disorder in which genetic predisposition significantly influences disease susceptibility. Women possessing a first-degree relative afflicted by endometriosis are confronted with an increased risk of up to twofold in comparison to those lacking a familial history (26% vs. 12%, *p* < 0.01) [12]. Large-scale genome-wide association studies (GWAS) have discerned numerous risk loci, particularly in proximity to genes implicated in immune regulation, hormone signaling, and inflammation, that contribute to the heritability of the condition, estimated to be approximately 10–12% across various populations [13]. Mendelian randomization analyses additionally indicate that particular inflammatory pathways, such as IL-6 signaling, may mediate this genetic risk [14].

b. Menstrual Cycle Characteristics

The premature onset of menstruation, commonly referred to as menarche, occurring before the age of 12, has been correlated with an elevated risk of developing moderate-to-severe endometriosis, as evidenced by a systematic review and meta-analysis of 18 case–control studies encompassing over 13,000 women [15]. Although the comprehensive analysis revealed a statistically non-significant increase in risk, the association attained significance when limited to studies exhibiting enhanced control over confounding variables. In addition to the occurrence of early menarche, menstrual characteristics such as short cycles and heavy or prolonged bleeding have also been associated with an increased risk of endometriosis. A meta-analysis of 11 case–control studies substantiated that women with cycle durations of 27 days or less encountered a markedly elevated risk [16], while extended cycles appeared to confer a protective effect—a conclusion corroborated by prior epidemiologic research [17]. A multicenter case–control study further illustrated that early menarche, abbreviated cycles, and adverse menstrual experiences—particularly among women whose mothers experienced dysmenorrhea—significantly heightened the risk of endometriosis [18], a conclusion that has been reiterated by more recent findings accentuating the necessity for early assessment of dysmenorrhea in adolescents [19].

c. Reproductive History

Researchers have identified nulliparity as a contributing factor to endometriosis, likely due to prolonged estrogen exposure and lack of pregnancy-related hormonal shifts [20,21,22].

d. Anatomical and Medical Conditions

Anatomical irregularities and particular medical conditions affect the predisposition to endometriosis. Among the most extensively recognized mechanisms is retrograde menstruation, a phenomenon characterized by the reverse flow of menstrual blood through the fallopian tubes into the pelvic cavity, thereby transporting viable endometrial cells. These cells may subsequently adhere to pelvic organs and instigate the formation of lesions. Although retrograde menstruation occurs in as many as 90% of menstruating women, only a small fraction (~10%) develop endometriosis, suggesting that additional biological factors must be implicated [23]. Classic experimental models involving baboons further corroborate this mechanism. In a seminal investigation, D’Hooghe illustrated that retrograde menstruation coupled with peritoneal inflammation was adequate to elicit endometriotic lesions in non-human primates, thereby reinforcing its causal association with human disease [24]. The foundational notion can be traced back nearly a century to Sampson’s hypothesis that menstrual dissemination through venous or lymphatic pathways could result in “metastatic” endometriosis, particularly at distant or extra-pelvic locations [25]. Nonetheless, anatomical risk factors such as obstructions to uterine outflow (e.g., imperforate hymen, cervical stenosis) and Müllerian anomalies (OMA) (e.g., septate or bicornuate uterus) exacerbate retrograde flow and intensify pelvic inflammation, thereby creating a conducive environment for ectopic implantation. These mechanical factors operate synergistically in conjunction with biological susceptibilities. This association is supported by meta-analytic data showing significantly higher prevalence of endometriosis in patients with obstructive OMA compared to non-obstructive cases, affirming the role of retrograde menstruation as a facilitating factor in the disease’s pathogenesis [26]. Further, anatomical reviews have emphasized that such obstructions increase intrauterine pressure and menstrual backflow, which may potentiate pelvic implantation and chronic inflammation [27]. Concurrently, emerging theories accentuate the roles of stem cell migration, coelomic metaplasia, and epigenetic dysregulation as plausible, and occasionally overlapping, mechanisms that elucidate instances not attributable to retrograde menstruation in isolation [28].

e. Body Mass Index (BMI)

Investigations have consistently demonstrated an inverse correlation between body mass index (BMI) and the likelihood of developing endometriosis, particularly regarding its more severe subtypes. The Nurses’ Health Study II, a prospective cohort study spanning 20 years, revealed that a lower BMI at the age of 18 and diminished waist-to-hip ratios were significantly associated with an elevated risk of endometriosis, indicating a potential connection between leanness and susceptibility to the disease [29]. A meta-analysis further corroborated this inverse relationship, illustrating that for every 5 kg/m^2^ increase in BMI, the risk of endometriosis was reduced by 33% [30]. These findings are substantiated by a case–control study involving 476 women, which evidenced that patients with deep infiltrating endometriosis (DIE) and ovarian endometriomas exhibited markedly lower BMIs, particularly those under 18.5, when compared to matched controls, with a threefold increased risk noted for DIE [31]. Furthermore, an Australian national cohort study indicated that women who experienced weight gain after the ages of 18 to 23 had a diminished risk of surgically confirmed endometriosis, whereas those who were classified as overweight during that age period demonstrated a heightened risk of physician-suspected endometriosis, suggesting that the diagnostic methodology may impact the observed associations [32]. In summary, these studies imply that a low BMI may not only serve as a risk factor for the development of endometriosis but may also be associated with the severity of the disease and the diagnostic pathways utilized.

f. Hormonal Factors

The development and persistence of endometriosis depend heavily on estrogen, which causes ectopic lesion proliferation while also stimulating inflammatory responses and disrupting normal hormonal regulation [33,34]. Research demonstrates that endometriotic lesions maintain high estrogen levels through local synthesis despite low systemic estrogen concentrations. The increased expression of aromatase (CYP19A1) allows endometriotic lesions to produce estrogen autonomously, a feature absent in healthy endometrial tissue. This local estrogen environment is further amplified by elevated activity of steroidogenic acute regulatory protein (StAR) and reduced expression of 17β-hydroxysteroid dehydrogenase type 2 (17β-HSD2)—an enzyme responsible for inactivating estradiol. These molecular alterations create a localized pro-estrogenic microenvironment that promotes lesion survival and progression [35,36,37]. Estrogen exerts its cellular functions by interacting with estrogen receptors ERα and ERβ. The expression levels of ERβ surpass those of ERα in endometriotic tissue, which creates a receptor dominance that supports pro-inflammatory and anti-apoptotic conditions [38]. ERβ promotes gene expression related to inflammation but reduces progesterone receptor levels, which creates progesterone resistance that characterizes endometriosis [39]. An imbalance in receptor levels triggers the production of prostaglandin E2 (PGE2), which acts as a powerful trigger for pain transmission and promotes both new blood vessel formation and immune response in abnormal tissue growth. Endometriosis treatments target the reduction in estrogen production or the interruption of its downstream pathways. Conventional therapeutic modalities, including combined oral contraceptives, gonadotropin-releasing hormone (GnRH) agonists, and progestins, primarily function by inhibiting ovulation and creating a hypoestrogenic milieu, which substantially diminishes lesion activity and mitigates symptoms such as pelvic pain and dysmenorrhea [40,41]. Although these interventions are efficacious for the management of symptoms, their prolonged application is frequently constrained by adverse effects, including osteopenia, vasomotor symptoms, and mood disturbances, particularly in the context of GnRH agonists [8,42]. Progestins such as dydrogesterone and dienogest generally exhibit a more favorable tolerability profile, rendering them appropriate for extended use among numerous patients; however, even these agents may result in deleterious effects in a minority of individuals [43] (Schweppe, 2009). Although medical progress has been made, scientists still lack complete comprehension of how estrogen functions at the molecular level within endometriosis. Researchers actively explore the unique roles and tissue distribution of ERα and ERβ along with their interactions with progesterone receptors and inflammatory pathways [38].

g. Immune System Dysfunction

The immune system plays a pivotal role in the development and progression of endometriosis. Both innate and adaptive immune responses contribute by driving inflammation, causing immune cell dysfunction, and altering cytokine production, which together create a chronic inflammatory environment that supports the survival and growth of ectopic endometrial tissue. In endometriosis, immune dysfunction impairs the clearance of these misplaced cells, allowing them to implant and proliferate outside the uterus—a process normally prevented by effective immune surveillance [44,45]. The peritoneal environment in affected women is marked by persistent inflammation, characterized by elevated levels of inflammatory cytokines such as interleukin-6 (IL-6) and tumor necrosis factor-alpha (TNF-α), alongside increased angiogenic factors that promote lesion maintenance and expansion [46,47]. Additionally, natural killer (NK) cell cytotoxic activity is markedly reduced, weakening the innate immune system’s ability to eliminate aberrant cells [44]. Furthermore, T-helper (Th) cell imbalance plays a critical role in the immunopathogenesis of endometriosis. In healthy immune responses, a balance exists between Th1 and Th2 cells, but in endometriosis, a shift toward a Th2-dominant profile has been observed [48]. This Th2 predominance is characterized by elevated levels of cytokines such as interleukin-4 (IL-4) and interleukin-10 (IL-10) in the peritoneal fluid of affected individuals, promoting anti-inflammatory and pro-angiogenic conditions that support ectopic tissue survival [48,49]. In addition, TH17 cells are implicated in the pathophysiology of endometriosis, characterized by dysregulation of the IL-23/TH17 axis. In endometriosis, IL-23 drives the differentiation of TH17 cells towards a pathogenic profile, leading to increased secretion of proinflammatory cytokines like IL-17. This dysregulation correlates with disease severity and contributes to key features of endometriosis, including lesion proliferation, vascularization, and inflammation. Studies show increased IL-23 levels in patient plasma and altered gene expression in ectopic and eutopic endometrial samples [50,51]. Concurrently, regulatory T cells (Tregs), which are essential for maintaining peripheral tolerance, show altered frequency and suppressive function in endometriosis, impairing immune control over ectopic lesions [52,53]. One study found that women with advanced endometriosis had significantly higher percentages of CD25+FOXP3+ Treg cells in peritoneal fluid compared to those with early endometriosis and control subjects [54]. This suggests that Treg cells may contribute to the survival and implantation of ectopic endometrial lesions by impairing the immune response against these cells, thus facilitating the progression of endometriosis. The cumulative effect of increased Th2 and Th17 activity alongside Treg dysfunction facilitates immune evasion and chronic inflammation, contributing to lesion persistence and disease progression.

Moreover, endometriosis shares several features with autoimmune diseases. Patients commonly exhibit increased autoantibody production and a higher prevalence of autoimmune disorders such as systemic lupus erythematosus and rheumatoid arthritis, suggesting overlapping pathways of immune dysregulation [55,56]. This autoimmune-like profile underscores the complex interplay between immune dysfunction and the pathogenesis of endometriosis.

h. Environmental Influences

Humans exposed to endocrine-disrupting chemicals (EDCs) present in pesticides and industrial contaminants, along with polymers and heavy metals, develop multiple health problems such as reproductive dysfunctions, metabolic disorders, and hormone-related cancers. The body’s endocrine system faces disruption from these substances through mechanisms that mimic hormones or block hormone production and signaling pathways, which can lead to harmful health effects [57].

Current research shows that EDCs potentially lead to both the origin and development of endometriosis by disrupting hormonal balance and triggering abnormal immune responses and inflammation. The estrogen-reliant nature of endometriosis suggests that when EDCs such as bisphenol A (BPA) and phthalates disrupt or imitate estrogenic functions, they could worsen the disease’s development and intensity through abnormal cell growth and hormonal imbalance [57].

The metabolic disorders that relate to EDC exposure amplify both inflammation and estrogen production, thereby worsening endometriosis symptoms [58]. Endometriosis involves biological pathways that overlap with hormone-sensitive cancers, and exposure to dioxins and PCBs, alongside other EDCs linked to these cancers, could increase endometriosis risk [59].

Prenatal exposure to EDCs shows a link to cognitive and behavioral abnormalities that suggest that early-life exposure leads to ongoing neurological and pain-related effects connected to endometriosis [60].

Research supports that EDC exposure contributes to both the development and worsening of endometriosis by interfering with hormonal balance, metabolic systems, immune defense mechanisms, and brain health.

## 3. Non-Hormonal Targets for Endometriosis

Treatment for endometriosis aims to reduce symptoms while slowing disease progression and improving fertility, when necessary, through personalized therapeutic plans.

Hormonal treatments, including combined oral contraceptives, progestins, GnRH agonists/antagonists, and aromatase inhibitors, form the first line of medical management to decrease menstruation and block endometrial growth [61]. NSAIDs, alongside other non-hormonal options, serve primarily as analgesic treatments [62,63].

When medication fails to produce desired outcomes or fertility preservation is not necessary, physicians consider surgical options such as conservative laparoscopic surgery and hysterectomy [33]. Affected individuals gain reproductive abilities through assisted reproductive technologies, including in vitro fertilization (IVF) [64].

New therapeutic approaches target hormonal and inflammatory pathways, while lifestyle changes, along with complementary treatments like dietary adjustments and physical exercise, provide additional symptom relief [56,65].

Hormonal treatment options are common in endometriosis management but inappropriate for patients who wish to become pregnant or those who experience negative treatment effects. The search for non-hormonal treatments that target the basic biological processes of endometriosis is gaining popularity.

Endometriosis represents a complex disorder that encompasses diverse molecular and cellular pathways that extend beyond hormonal influences. A comprehensive understanding of these non-hormonal mechanisms has facilitated the exploration of novel targeted therapeutic approaches. The following sections delineate some of these pathways, providing pertinent background information and elucidating their significance in the pathophysiology of endometriosis.

### 3.1. Inflammatory Pathway

Chronic inflammation serves as a fundamental element in the onset and advancement of endometriosis. Elevated concentrations of pro-inflammatory cytokines, including IL-1β, IL-6 [66], and TNF-α [67,68], contribute to the development of lesions, the experience of pain, and the occurrence of fibrotic alterations. Immune cells, such as neutrophils and macrophages [69], exhibit heightened activity in the context of endometriosis [70,71], while chemokines like RANTES (CCL5) further intensify inflammation by recruiting additional immune cells [72]. Dysregulation of T cell populations fosters a pro-inflammatory milieu that facilitates lesion viability [73,74,75]. The accumulation of regulatory T cells (Tregs) within lesions and peritoneal fluid promotes immunosuppression and fibrogenesis through cytokines such as TGF-β1 [73,74,76,77]. These inflammatory mechanisms are intricately linked with hormonal signaling and stress responses within the peritoneal cavity [78], thereby rendering anti-inflammatory agents (e.g., vitamin D receptor agonists [79], resveratrol [80,81]) as promising candidates for non-hormonal therapeutic interventions. In this area, therapeutic strategies are being evaluated which target inflammation through nuclear factor kappa B pathways and immune cell regulation along with cytokine modulation. JNK inhibitors like bentamapimod are immunomodulatory agents that have shown promising results in clinical studies for reducing endometriosis-associated pain [82,83,84].

Research demonstrates that not all pre-existing inflammatory conditions follow the same mechanisms or contribute uniformly to disease severity, emphasizing gaps in our understanding of inflammation’s diverse roles across gynecologic disorders, which underlines the importance of additional research into distinct inflammatory processes [85].

### 3.2. Immune Dysregulation Pathways

Immunological dysfunction constitutes a defining characteristic of endometriosis, typified by diminished natural killer (NK) cell activity and an increase in Tregs [86]. NK cells in individuals afflicted with endometriosis demonstrate compromised cytotoxic capabilities due to heightened expression of inhibitory receptors like CD158a and elevated rates of apoptosis, thereby impairing their effectiveness in eliminating ectopic endometrial tissue [87]. The accumulation of Tregs within peritoneal fluid and lesions engenders an immunosuppressive environment that facilitates the persistence and proliferation of lesions [52]. Therapeutic strategies aimed at reinstating immune surveillance and diminishing immune tolerance are currently under investigation as viable non-hormonal interventions.

### 3.3. Epithelial-Mesenchymal Transition (EMT) and Fibrosis Pathways

Epithelial-mesenchymal transition (EMT) constitutes a pivotal biological process in endometriosis, characterized by the detachment of endometrial cells from their adhesive properties and the acquisition of migratory and invasive characteristics. Transforming growth factor-beta (TGF-β), particularly TGF-β1, plays a central role in the induction of EMT, leading to the upregulation of mesenchymal markers and transcription factors in endometriotic stromal cells [88]. Additionally, TGF-β1 promotes fibrosis by converting fibroblasts into myofibroblasts, thereby enhancing collagen synthesis and extracellular matrix accumulation. This fibrotic response culminates in the formation of adhesions and scarring, which contributes to chronic pelvic pain and infertility [89,90]. Targeting TGF-β signaling and associated fibrotic pathways presents a promising avenue for non-hormonal therapeutic intervention.

### 3.4. Angiogenesis Pathways

Angiogenesis, defined as the process of forming new blood vessels, is crucial for the sustenance and proliferation of endometriotic lesions [91,92]. Vascular endothelial growth factor (VEGF) exhibits significantly elevated levels in the peritoneal fluid and lesions of women diagnosed with endometriosis [93], thereby fostering an angiogenic microenvironment that sustains lesion maintenance and progression [94]. Anti-angiogenic therapies that target VEGF or its associated signaling pathways represent promising non-hormonal strategies aimed at curtailing lesion growth and mitigating disease progression. Anti-angiogenic drugs like dopamine agonists cabergoline and quinagolide have proven effective in multiple clinical trials [82,84,94].

### 3.5. Oxidative Stress Pathway

Oxidative stress is a critical factor in the pathophysiology of endometriosis, as it disrupts the equilibrium between reactive oxygen species (ROS) and the body’s antioxidant defenses. This dysregulation results in the accumulation of ROS within the peritoneal fluid, leading to cellular injury and the initiation of inflammatory responses [95]. The presence of iron overload stemming from retrograde menstruation further amplifies ROS generation, thereby intensifying oxidative stress and inflammatory processes [95,96,97]. These pathological states activate various signaling cascades, including MAPK and PI3K/AKT/mTOR, which promote cellular survival and proliferation [95,98]. The restoration of redox homeostasis through the application of antioxidants, alongside the targeting of oxidative stress pathways, is currently being explored as a potential non-hormonal therapeutic strategy [84].

Several antioxidant supplements have shown promise in ameliorating oxidative stress and inflammation associated with endometriosis:

- Silymarin: Derived from milk thistle, silymarin exhibits potent antioxidant and anti-inflammatory properties, reducing lipid peroxidation and modulating inflammatory cytokine production [99,100]. Silymarin has been shown to significantly reduce interleukin-6 levels, the size of endometrioma lesions, and pain symptoms in women with endometriosis, according to a randomized, double-blind, placebo-controlled trial. In this study, 70 women received either 140 mg of silymarin or a placebo twice daily for 12 weeks, resulting in significant improvements in endometrioma volume (*p* = 0.04), IL-6 levels (*p* = 0.002), and pain (*p* < 0.001), although quality of life and sexual function did not improve substantially [101].
- N-acetylcysteine (NAC): NAC serves as a precursor for glutathione synthesis, the body’s main intracellular antioxidant. Studies have demonstrated that NAC reduces lesion size and oxidative stress markers in endometriosis models and improves clinical symptoms [102,103].
- Omega-3 Fatty Acids: Omega-3 polyunsaturated fatty acids (PUFAs) exert anti-inflammatory effects by modulating eicosanoid pathways, reducing pro-inflammatory prostaglandins and cytokines. PUFAs have been studied for their potential therapeutic effects on endometriosis [104]. These fatty acids, particularly eicosapentaenoic acid (EPA) and docosahexaenoic acid (DHA), are known to modulate inflammatory pathways, which could theoretically alleviate symptoms associated with endometriosis, such as pelvic pain. However, the evidence from clinical trials remains inconclusive, with some studies showing benefits in animal models but limited evidence of significant effects in human trials. A pilot trial indicated the feasibility of conducting larger studies to evaluate the efficacy of omega-3 PUFAs for endometriosis-associated pain, although no significant pain reduction was observed in the short term [105].
- Curcumin: The active compound in turmeric, curcumin, is a powerful antioxidant and anti-inflammatory agent [106]. It inhibits NF-kB activation and reduces cytokine production, with preclinical studies indicating its potential to reduce lesion growth and pain [106]. Despite promising preclinical results, a clinical trial indicated that curcumin did not significantly alleviate pain or improve the quality of life in women with endometriosis, suggesting that its efficacy in humans may be limited or require further investigation [107].

While these supplements are promising, further large-scale clinical trials are necessary to establish standardized dosing and long-term efficacy.

### 3.6. Genetic and Epigenetic Pathways

Genetic predisposition and epigenetic alterations significantly contribute to the heterogeneity and complexity observed in endometriosis. Genome-wide association studies have elucidated several susceptibility genes, such as WNT4, GREB1, and FN1, which impact cellular proliferation and hormonal responsiveness [108,109]. Epigenetic modifications, including DNA methylation and histone alterations, influence gene expression patterns, resulting in progesterone resistance and chronic inflammation [110]. Recent meta-analyses have identified specific genetic polymorphisms correlated with endometriosis, thereby suggesting the feasibility of developing genetic screening tools for early diagnosis and risk stratification. Additionally, epigenetic therapies hold promise as novel approaches for reversing aberrant gene expression in endometriotic tissues [111].

Non-coding RNAs (ncRNAs), including microRNAs (miRNAs) and long non-coding RNAs (lncRNAs), regulate gene expression at the post-transcriptional level and have been implicated in the modulation of inflammatory, immune, and fibrotic pathways [112,113]. Although emerging evidence highlights their potential as diagnostic biomarkers and therapeutic targets, the current literature remains limited and primarily preclinical. Due to the nascent stage of research, ncRNA-based therapies are not yet widely discussed in clinical contexts and thus are only briefly mentioned here, pending further validation.

### 3.7. Microbiota Dysbiosis

Emerging research indicates that dysbiosis of the microbiota, perturbations in the composition of gastrointestinal and vaginal microbial communities, contributes significantly to the pathophysiological mechanisms underlying endometriosis [114]. Dysbiosis may engender immune activation, promote chronic inflammatory responses, and facilitate the persistence of lesions via various interconnected pathways. Importantly, alterations in the gut microbiota can compromise the integrity of the mucosal barrier, permitting the translocation of microbial constituents such as lipopolysaccharides (LPS), which, in turn, activate toll-like receptor (TLR)-mediated inflammatory pathways [115]. This inflammatory response may exacerbate the proliferation of endometriotic lesions. Moreover, the estrobolome, a component of the microbiome responsible for estrogen metabolism, can modulate systemic estrogen concentrations and potentially perpetuate the hormonal milieu conducive to ectopic endometrial tissue [114]. Recent clinical investigations have demonstrated that individuals with endometriosis frequently exhibit an elevated prevalence of Bacteroidetes and a diminished presence of Firmicutes, thereby reflecting a state of gut microbial dysbiosis [114]. In the vaginal microbiome, dysbiosis is characterized by a reduction in beneficial *Lactobacillus* species and an increase in opportunistic genera such as *Gardnerella* and *Desulfovibrio*, which may compromise local immune responses and instigate inflammation associated with lesions [116]. These findings elucidate potential pathways for therapeutic interventions aimed at reinstating microbial equilibrium, including the application of antibiotics (e.g., metronidazole, chloramphenicol), probiotic formulations, and innovative microbiome-centric biotherapeutics such as FP-300. While these strategies exhibit considerable potential, further empirical research is imperative to substantiate their long-term effectiveness and to assimilate them into individualized treatment regimens for endometriosis.

### 3.8. Metabolic Reprogramming

Endometriotic lesions are distinguished by considerable metabolic reprogramming, exhibiting numerous characteristics akin to those observed in neoplastic tissues. This encompasses a transition towards aerobic glycolysis (often referred to as the Warburg effect), diminished oxidative phosphorylation, and compromised mitochondrial functionality, collectively sustaining the viability of lesions and facilitating cellular proliferation within hypoxic peritoneal environments [117,118].

Recent investigations have revealed that endometriotic cells exhibit augmented glycolytic flux, heightened expression of glycolytic enzymes (such as hexokinase-2 and lactate dehydrogenase A), and compromised mitochondrial bioenergetics—culminating in an acidic, pro-inflammatory microenvironment that fosters angiogenesis and immune evasion [119]. These adaptations not only mirror oncogenic metabolism but also contribute to lesion resilience and recurrence.

A particularly promising strategy for intervention focuses on these metabolic vulnerabilities. Dichloroacetate (DCA), an inhibitor of pyruvate dehydrogenase kinase (PDK), reestablishes mitochondrial oxidative metabolism by facilitating the conversion of pyruvate to acetyl-CoA. This shift mitigates lactate accumulation and curtails aberrant cellular proliferation. Preclinical investigations—including in vitro coculture models and murine studies—indicate that DCA may inhibit the proliferation of endometriotic stromal cells by recalibrating mitochondrial activity and downregulating reliance on glycolysis [117,120]. However, human clinical trials remain sparse and primarily exploratory.

Moreover, mitochondrial dysfunction within endometriotic cells may exacerbate oxidative stress and hinder apoptotic signaling, thereby contributing to the persistence of lesions and resistance to hormonal therapies. These observations underscore the potential utility of metabolic modulators such as DCA or mitochondrial-targeted antioxidants as complementary agents to hormonal therapies in endometriosis phenotypes that exhibit resistance [121].

### 3.9. Neuropathic Pain Modulators

#### 3.9.1. Neuroinflammation and Central Sensitization

Chronic pelvic pain (CPP) associated with endometriosis is increasingly recognized as a manifestation of neuroinflammation and central sensitization, a condition wherein the central nervous system (CNS) exhibits heightened responsiveness to nociceptive stimuli. The recurrent nociceptive signaling emanating from endometriotic lesions precipitates neuroplastic alterations within spinal and cerebral regions, particularly highlighting glial activation within the thalamus, hippocampus, and cortex. This phenomenon has been substantiated in a 2025 murine investigation, wherein multiple lesion inductions emulating retrograde menstruation resulted in sustained hyperalgesia, augmented microglial and astrocytic activation, elevated levels of proinflammatory cytokines (IL-1β, TNF-α), and increased expression of pain-related neuropeptides such as CGRP and substance P [122]. These observations elucidate the significance of neuroinflammation in exacerbating pain even in the absence of active lesions. To mitigate this condition, neuromodulatory interventions, including gabapentinoids, serotonin–norepinephrine reuptake inhibitors (SNRIs), and tricyclic antidepressants (TCAs), are employed in clinical settings to diminish neuronal hyperexcitability and augment descending inhibitory mechanisms [122]. Investigational modalities such as NMDA receptor antagonists (for instance, ketamine) are currently under examination for their potential to attenuate excitatory glutamatergic transmission and alleviate pain hypersensitivity. Moreover, non-pharmacological methodologies, including transcutaneous electrical nerve stimulation (TENS) and cognitive behavioral therapy (CBT), also play a role in modulating neural pain pathways and enhancing emotional pain processing [123]. Furthermore, dysfunction of the endocannabinoid system (ECS) has been delineated in endometriosis, characterized by aberrant expression patterns of CB1 and CB2 receptors within both eutopic and ectopic endometrial tissues. This dysregulation contributes to diminished analgesic efficacy and exacerbated inflammatory signaling, rendering the ECS a compelling target for therapeutic intervention. Cannabinoid-based treatments, such as cannabidiol (CBD) and investigational compounds from Gynica, seek to restore endocannabinoid homeostasis and attenuate neuroinflammatory signaling with minimal hormonal repercussions [122]. Collectively, these therapeutic modalities comprise a nascent class of neuropathic pain modulators in endometriosis, reflecting an evolving paradigm focused on addressing the neurobiological underpinnings of chronic pain beyond mere lesion suppression.

#### 3.9.2. Endocannabinoid Dysfunction

The endocannabinoid system (ECS), encompassing CB1 and CB2 receptors, endocannabinoids (such as anandamide and 2-AG), and their associated metabolizing enzymes, plays an essential role in the modulation of inflammatory processes, nociception, and immune responses. In the context of endometriosis, perturbations in ECS signaling have been documented in both eutopic and ectopic endometrial tissues, resulting in alterations in pain perception and immune activation.

Specifically, the expression of the CB1 receptor is frequently diminished in endometriotic lesions, which undermines the ECS’s inherent anti-nociceptive capabilities and facilitates the manifestation of chronic pelvic pain. Dysregulation of CB2 receptors, predominantly located on immune cells, is also noted, potentially aggravating localized inflammation and cytokine secretion. These modifications engender a neuroinflammatory milieu and sensitized neural environment that are conducive to chronic pain [124].

Cannabinoid-based therapies have emerged as promising non-hormonal alternatives for alleviating symptoms. Cannabidiol (CBD)—a cannabinoid devoid of psychoactive effects—has demonstrated potential in both preclinical and patient-reported studies for mitigating endometriosis-associated pain through the modulation of peripheral inflammation and central sensitization. CBD acts by inhibiting FAAH (fatty acid amide hydrolase), thereby prolonging the action of anandamide and enhancing CB1/CB2 signaling pathways. Furthermore, formulations developed by companies such as Gynica are undergoing clinical evaluation for targeted delivery of cannabinoids via vaginal or intrauterine routes, with the objective of achieving symptom control while minimizing hormonal side effects [124,125].

In addition to pain alleviation, modulation of the ECS may also have implications for reproductive hormone pathways and immune responses, thereby augmenting its therapeutic potential in a condition characterized by dysregulation of both endocrine and inflammatory processes [126].

Table 1 summarizes potential non-hormonal targets for endometriosis, while Table 2 outlines corresponding non-hormonal therapeutic agents.

## 4. Off-Label Selected Non-Hormonal Therapies

### 4.1. The Renin–Angiotensin System

The renin–angiotensin system (RAS) plays a complex and multifunctional part in both the development and progression of endometriosis through its roles in promoting angiogenesis, inflammation, and fibrosis, which are key processes involved in the disease [162].

- *Mechanism of Action*

Angiogenesis Pathway

The primary effector of the renin–angiotensin system (RAS), angiotensin II (Ang II) stimulates the production of vascular endothelial growth factor (VEGF), which leads to increased angiogenesis. Endometriotic lesions rely on neovascularization for essential nutrients and oxygen delivery, which maintains their viability and stimulates their growth. Increased levels of angiotensin-converting enzyme (ACE) in endometriotic tissue elevate Ang II concentrations, which intensifies the tissue’s angiogenic response [162,172].

Inflammatory Pathway

Ang II functions as a pro-inflammatory mediator through the stimulation of cytokine release, including tumor necrosis factor-alpha (TNF-α), interleukin-6 (IL-6), and interleukin-1 beta (IL-1β). The inflammatory cytokines worsen the persistent inflammatory environment found in endometriosis, which leads to immune system dysfunction and allows ectopic endometrial cells to avoid immune destruction and remain in the peritoneal cavity [162].

Studies demonstrate that Ang II induces pro-inflammatory cytokine production through nuclear factor-kappa B (NF-κB) pathway activation, which results in increased TNF-α and IL-6 expression. Research has shown increased levels of IL-1β, IL-6, and TNF-α in tissues affected by endometriosis, which confirms the crucial role of these cytokines in the disease’s pathophysiology [173,174]. The inflammatory environment supports ectopic endometrial tissue implantation while also worsening endometriosis-related pain and infertility.

Fibrosis and EMT Pathway

Ang II initiates fibrosis and epithelial–mesenchymal transition (EMT) via activation of TGF-β signaling. This leads to increased extracellular matrix deposition and fibrotic adhesion formation, contributing to chronic pelvic pain and infertility [88,175]. The Smad signaling pathway mediates this TGF-β-driven process, enhancing the invasive potential of endometriotic lesions [176]. The process helps sustain endometriotic lesions and enables their invasive behavior while also forming fibrotic connections in the pelvic area [177].

Genetic Pathway

Endometriosis involves pathological processes including angiogenesis and fibrosis, where RAS takes part through regulatory means, with ACE serving as an essential component. A rigorous meta-analysis examined the link between endometriosis risk and genetic variations in the ACE and PAI-1 genes through eleven studies consisting of 1486 cases and 1598 controls. The genetic variants ACE 2350A/G, ACE -240A/T, and PAI-1 4G/5G demonstrated significant links to increased susceptibility to endometriosis [178]. The findings highlight a possible genetic susceptibility to endometriosis, which involves components of both the RAS and the fibrinolytic systems [179].

- *Preclinical and Clinical Evidence:*

Expression Studies: Both angiotensin II type 1 (AT1) and type 2 (AT2) receptors are expressed in endometrial tissues, exhibiting altered expression patterns in the context of endometriosis. RAS-related genes (AGT, AGTR1, ACE1, and ACE2) are identified in most endometrial samples, signifying extensive expression of RAS components [162].

Angiogenesis: Dysregulated expression of AT1R and AT2R is correlated with vascular alterations in the endometrium, influencing angiogenic activities pertinent to endometriosis [175].

Physiological Studies: Women diagnosed with endometriosis demonstrate aberrant RAS activity, characterized by reduced plasma renin levels, elevated aldosterone-renin ratios, and increased systolic blood pressure [162].

Therapeutic Implications: RAS inhibitors, such as angiotensin-converting enzyme inhibitors (ACEIs) and angiotensin receptor blockers (ARBs), are being investigated as prospective non-hormonal therapeutic options, demonstrating advantageous outcomes such as diminished angiogenesis, suppression of inflammatory cytokines (IL-6, TNF-α), and reduction in fibrosis and adhesion formation [162].

RAS research indicates its potential role in various gynecological disorders because changes in Ang II activity appear to affect disease progression [180].

Research findings demonstrate that Ang II and its receptors AT1R and AT2R are present in key reproductive tissues such as the ovaries, uterus, and placenta, which indicates the existence of a localized RAS system [175,181,182,183]. Furthermore, imbalance between Ang II and Ang (1–7) levels correlates with reproductive disorders that can negatively impact fertility [181].

Researchers now study RAS inhibitors, including ACEIs and ARBs, as potential treatments for endometriosis because these drugs target the system responsible for angiogenesis, inflammation, and fibrosis. These medical compounds deliver several beneficial effects, which include the following:

✓ Reduce VEGF-mediated angiogenesis, thereby limiting lesion vascularization and growth [164].
✓ Suppress inflammatory cytokine production, including IL-6 and TNF-α, which may contribute to pain reduction [164].
✓ Decrease fibrosis and adhesion formation, potentially improving reproductive outcomes by mitigating tissue remodeling and scarring [184].

The angiotensin pathway plays a crucial role in advancing endometriosis development through angiogenic processes as well as inflammatory and fibrotic mechanisms. The engagement of ACE inhibitors and ARBs in the angiotensin pathway indicates potential for these drugs to serve as non-hormonal treatment alternatives for endometriosis management.

Evidence of RAS significance in endometriosis is presented in Table 3.

- *Limitations:*

Translational Gap: Despite the comprehensive documentation of RAS components and their associated roles in tissue and animal model studies, there is a notable deficiency of large-scale clinical trials evaluating the efficacy of RAS inhibitors in the management of endometriosis.

Complexity: The intricate role of RAS in angiogenesis, inflammation, and fibrosis presents significant challenges in the formulation of targeted therapeutic strategies.

Genetic Diversity: Variability in genetic susceptibility suggests that not all patients may experience equivalent benefits from RAS-targeted therapeutic interventions.

- *Safety Profile:*

General Tolerability: While RAS inhibitors are widely used in other conditions (e.g., hypertension), specific safety data in endometriosis are limited in the literature. However, their established safety in other populations suggests a favorable profile.

Potential Concerns: Hypotension and electrolyte disturbances are possible with RAS inhibitors, but no significant adverse events have been reported in endometriosis studies to date [175,181,182,183,184].

- *Future Directions:*

Clinical Trials: Large, well-designed clinical trials are needed to evaluate the efficacy and safety of RAS inhibitors in endometriosis.

Personalized Medicine: Research into genetic markers may help identify patients most likely to benefit from RAS-targeted therapies.

Mechanistic Studies: Further studies are needed to clarify the specific roles of RAS components in endometriosis pathogenesis and progression.

### 4.2. Antibiotic Therapy in Endometriosis: Emerging Insights

Antibiotics as endometriosis treatments represent a novel research field that examines their potential effects on improving fertility outcomes while slowing disease progression.

- *Mechanism of Action:*

Treatment of Chronic Endometritis (CE): Chronic inflammatory endometriosis frequently coexists with chronic endometritis (CE), a condition implicated in recurrent implantation failure (RIF) and recurrent miscarriage. Clinical studies show that antibiotic treatment of CE improves pregnancy and live birth rates in affected women [158,189].

Microbiota Modulation: Antibiotics modify gut and vaginal microbiota, potentially affecting endometrial immune status and endometriosis progression. Studies suggest that restoration of healthy microbiota after antibiotic therapy, possibly with probiotics, supports better reproductive outcomes [190].

Immunological Effects: Antibiotic therapy reduces pro-inflammatory cytokines, such as IL-6 and TNF-α, in CE and endometriosis, mitigating the chronic inflammatory environment that fosters lesion survival [162,191].

Reduction in Lesion Growth: Animal and human data suggest that antibiotics such as metronidazole or lincomycin can reduce the volume and inflammation of endometriotic lesions, likely through microbiota-mediated immune modulation [191].

- *Preclinical and Clinical Evidence:*

Improved Reproductive Outcomes: Women who receive antibiotic therapy for CE experience higher pregnancy and live birth rates when dealing with RIF. The 2023 study titled “The Impact of Antibiotic Treatment on the Obstetric Prognosis of Patients with Chronic Endometriosis”, together with research by Domiciano et al. (2022), supports this association [153]. A systematic review and meta-analysis [154] found that women who resolved CE experienced significantly better ongoing pregnancy and live birth outcomes versus women who continued to experience persistent CE.

Route of Administration: The 2022 study by Luncan et al. showed that intrauterine antibiotic infusion cured CE and improved fertility outcomes for in vitro fertilization (IVF) patients better than oral antibiotic treatment [155].

Animal Studies: Antibiotic treatment in animal models reduces lesion growth and inflammation and alters gut microbiota composition. For instance, Chadchan et al. (2019) found that metronidazole decreased inflammation levels and lesion dimensions, indicating that gut microbiota affects disease advancement [156].

Targeted and Combined Therapies: Lu et al. (2022) revealed how combining vaginal microbiota transplantation with NF-κB-targeted antibiotics successfully slowed disease progression in mice [157]. The use of antibiotics leads to lower serum CA-125 levels, which tend to increase in endometriosis patients, suggesting an infectious aspect of the condition [158].

Immunological Impact: Research on animals demonstrates that antibiotic treatment lowers levels of important inflammatory cytokines, including IL-1β, IL-6, and TNF-α, which are associated with endometriosis development [157].

Microbiome Interactions: Symptom severity and progression of the disease are critically influenced by interactions between gut and genital microbiota. Salliss et al. 2021 demonstrate that microbiome imbalances worsen symptoms while antibiotics restore microbial equilibrium, which then lessens inflammation and hormone-related disease processes [159].

- *Limitations:*

Translational Gap: Most evidence for antibiotics in endometriosis comes from animal studies or from treating CE in the context of infertility; direct evidence for treating endometriosis itself in humans is limited.

Antibiotic Resistance: Repeated or inappropriate use may contribute to the development of resistant bacterial strains.

Microbiome Disruption: Antibiotics may disrupt healthy microbiota, potentially leading to other health issues.

Patient Selection: Not all patients with endometriosis have CE or microbiome imbalances, so broad application may not be appropriate.

- *Safety Profile:*

General Tolerability: Antibiotics are generally well-tolerated for short-term use in treating CE and related infections.

Potential Concerns: Risks include allergic reactions, gastrointestinal upset, and, with prolonged or repeated courses, possible disruption of beneficial microbiota and increased risk of antibiotic resistance [153,154,155,156,157,158,159].

Long-term Effects: The long-term safety of repeated or chronic antibiotic use for endometriosis is not yet established and requires further study [153,154,155,156,157,158,159].

- *Future Directions:*

Microbial Health: Further research should analyze the impact of prolonged antibiotic use on the body’s microbial communities.

Resistance Monitoring: Ongoing surveillance for antibiotic resistance is essential.

Mechanistic Studies: More studies are needed to clarify how antibiotics interact with endometriosis pathophysiology and the microbiome.

Personalized Medicine: Development of personalized treatment strategies that incorporate microbiome modulation and careful patient selection.

Clinical Trials: Well-designed, controlled clinical trials are needed to confirm the efficacy and safety of antibiotic therapy for endometriosis and to define optimal protocols.

## 5. Benefits and Limitations of Non-Hormonal Therapies

Benefits: Non-hormonal treatments provide viable options for women who cannot undergo hormonal therapy because of medical contraindications or personal choice, especially for those who wish to become pregnant [82,84].

Limitations: Most non-hormonal therapeutic approaches function through suppression instead of cure, and many remain in the preclinical or early clinical trial stages. The transition from preclinical discoveries to human trials continues to face major challenges, which lead to limited availability of effective treatments [82,83].

- *Challenges and Future Directions*

Research Gaps: Many potential pharmacological drugs have failed to advance past preclinical animal testing because of flawed study designs and the lack of appropriate experimental models [82,83].

Emerging Areas: The research of non-coding RNAs, along with bacterial infection implications such as Fusobacterium, for non-hormonal treatment strategies represents innovative therapeutic targets currently under investigation [192,193].

Need for Clinical Trials: The safety and effectiveness of these therapeutic modalities in humans need further validation through comprehensive and well-structured clinical trials [82].

## 6. Conclusions

Endometriosis represents a complex and heterogeneous pathological condition that significantly influences both the health and quality of life of women. The intricate and multifactorial origins of this ailment, encompassing genetic, hormonal, anatomical, and immunological dimensions, necessitate a comprehensive and integrative approach for effective diagnosis and treatment. While hormonal therapies remain the primary foundation of therapeutic management, their intrinsic limitations underscore an urgent requirement for viable non-hormonal alternatives. Advances in understanding the molecular and immunological pathways linked to endometriosis have propelled the development of innovative non-hormonal pharmacological treatments designed to target the fundamental etiological components of the condition, rather than solely alleviating its symptoms. Improved diagnostic methodologies, particularly non-invasive biomarker assays, hold the promise of reducing diagnostic delays and facilitating prompt interventions. Continuous research is essential to refine these strategies, ensure equitable healthcare access, and improve clinical outcomes for women affected by endometriosis on a global scale.

In this dynamic therapeutic environment, there is an emergence of a shift toward personalized, multi-targeted therapeutic strategies. Therapeutic agents such as cabergoline and cannabinoid-based therapies, currently undergoing late-phase clinical trials, exemplify promising non-hormonal alternatives with significant translational potential. When synergistically combined with anti-inflammatory, antioxidant, microbiota-modulating, or neuromodulatory therapies, these strategies may facilitate the development of customized treatment regimens that address the diverse biological mechanisms underlying the disease. Nevertheless, despite the increasing promise of these therapies, the majority remain in investigational stages, and their routine clinical application necessitates validation through rigorous, large-scale randomized controlled trials. This transition not only broadens therapeutic options but also signifies a change in thinking in the prospective management of endometriosis—characterized by precision, caution, and a patient-centered approach.

## Figures and Tables

**Table 1:** Potential non-hormonal targets in endometriosis.

Mechanism	Key Features	Therapeutic Approaches
Inflammatory	Cytokines (IL-1β, IL-6, TNF-α); immune cells; chemokines	Anti-inflammatory agents (e.g., NSAIDs, vitamin D agonists, resveratrol, curcumin, metformin, NAC)
Immune Dysregulation	NK cell dysfunction; regulatory T cells (Tregs); inflammasome (NLRP3)	Immune modulators (cabergoline, pentoxifylline), NK cell enhancers, NLRP3 inhibitors, JNK inhibitors
EMT and Fibrosis	TGF-β signaling; myofibroblast transformation; collagen deposition	Anti-fibrotic agents (RAS blockers like losartan), TGF-β pathway inhibitors, tissue remodeling modulators
Angiogenesis	VEGF, neovascularization, endothelial proliferation	Anti-angiogenic drugs (e.g., kinase inhibitors like VAL-301), VEGF inhibitors, cabergoline
Oxidative Stress	Reactive oxygen species (ROS); iron overload; lipid peroxidation	Antioxidants (NAC, silymarin, omega-3 fatty acids, curcumin), redox modulators
Genetic/Epigenetic	Susceptibility genes (e.g., WNT4, GREB1); DNA methylation; histone modifications	Epigenetic therapies (experimental), gene expression modulators, biomarker-based interventions (TTX334Dx)
Microbiota Dysbiosis	Altered vaginal/gut microbiome; bacterial contamination	Microbiome modulators (antibiotics like metronidazole and chloramphenicol; FP-300 bacterial therapies)
Metabolic Reprogramming	Altered glycolysis; lactate accumulation; mitochondrial dysfunction	Metabolic modulators (e.g., dichloroacetate)
Neuropathic Pain Modulators: Neuroinflammation/Central Sensitization	Central nervous system sensitization; persistent nociceptive signaling	Neuromodulators (gabapentinoids, antidepressants), CBT, TENS, NMDA antagonists (experimental)
Neuropathic Pain Modulators: Endocannabinoid Dysfunction	CB1/CB2 receptor alterations; pain signaling dysregulation	Cannabinoid-based therapies (CBD, Gynica formulations under clinical investigation)

**Table 2:** Non-hormonal therapies for endometriosis.

Mechanism	Therapy	Study Type	Model	Outcomes	References
Anti-inflammatory/ Antioxidant	Curcumin	Preclinical and clinical	Animal, Human	Preclinical efficacy; limited clinical benefit	[106,107]
	Metformin	Preclinical mainly	Cells, Animal, Human	↓ lesion size and inflammation; some human support	[127,128,129,130,131,132,133,134,135,136,137,138,139]
	N-acetylcysteine (NAC)	Preclinical and clinical	Animal, Human	↓ lesion size and oxidative stress; improved symptoms	[102,103]
	Omega-3 Fatty Acids	Pilot study	Animal, Human	Feasible; inconclusive short-term pain outcomes	[105]
	NSAIDs (e.g., ibuprofen)	Clinical/observational	Human	Mild pain relief; no disease-modifying effect	[140]
	Silymarin	RCT (n = 70)	Human	↓ IL-6 (*p* = 0.002), ↓ endometrioma (*p* = 0.04), ↓ pain (*p* < 0.001); no QoL improvement	[99,100,101]
Immunomodulatory/ Cytokine Inhibitors	Cabergoline (dopamine agonist)	RCT; multicenter (ongoing)	Human	Improved pain and QoL; fewer hormonal side effects; anti-angiogenic	[82,141,142,143,144]
	Celmatix JNK Inhibitor	Preclinical	Animal	Immunotherapy-induced lesion regression	[145,146]
	Kinase Inhibitors (e.g., VAL-301)	Preclinical, early clinical	Animal, Human	↓ angiogenesis and lesion growth; early clinical evaluation ongoing	[145,146,147]
	NLRP3 Inhibitors	Preclinical	Animal	↓ lesions and inflammatory markers	[148]
	Pentoxifylline	Pilot (animal, human)	Animal, Human	↓ inflammation in preclinical models; limited clinical support	[149,150]
	TNF-α Inhibitors (Infliximab, Etanercept, Adalimumab)	RCT and pilot studies	Animal, Human	Some efficacy in models; minimal clinical benefit	[151,152]
Microbiota-Targeting	Antibiotics (Metronidazole, Chloramphenicol)	Preclinical, clinical	Animal, Human	↓ inflammation and lesion growth and improve fertility outcomes, though human trial evidence remains limited	[153,154,155,156,157,158,159]
	FP-300 (Flightpath Bio)	Preclinical	Animal		[145]
Metabolic Modulators	Dichloroacetate	RCT (Phase 2 ongoing); preclinical	Animal, Human	↓ lactate production and lesion volume	[145,160]
Fibrosis and Tissue Remodeling	RAS Blockers (e.g., Losartan, Candesartan, Telmisartan)	Animal; pilot human	Animal, Human	↓ lesion burden, fibrosis, and pain; early human support	[161,162,163,164,165]
	Temple TTX334Dx	Preclinical, early clinical	Animal, Human	Biomarker-based targeting under development	[145]
Immune-Directed/ Targeted	FimmCyte (FMC2)	Preclinical (mouse); clinical planned 2025	Animal, Human	↓ disease burden in models; human trials pending	[145,160]
Neuropathic Pain Modulators: Neuroinflammation/ Central Sensitization	Gabapentinoids (Gabapentin, Pregabalin)	Clinical/observational	Human	↓ neuronal hyperexcitability; improved chronic pelvic pain	[166,167,168]
	SNRIs (e.g., Duloxetine), TCAs (e.g., Amitriptyline)	Clinical/observational	Human	↓ central pain sensitization; enhanced descending pain inhibition	[166,168]
	NMDA Receptor Antagonists (e.g., Ketamine)	Preclinical and early clinical	Animal, Human	↓ glutamate-mediated excitatory signaling; ↓ chronic pelvic pain	[169,170]
	JNK Inhibitors (e.g., SP600125)	Preclinical (cell-based models)	Human cells	↓ IL-1β-induced neurotrophins (NGF, BDNF); ↓ neuroinflammatory signaling	[171]
	P2X3 Antagonists (e.g., Gefapixant)	Preclinical/Phase I–II	Animal, Human	↓ afferent sensory hypersensitivity; under investigation for pelvic pain relief	[171]
Neuropathic Pain Modulators: Endocannabinoid Dysfunction	Cannabidiol (CBD)	Double-blind RCT (ongoing)	Human	Results pending on pain outcomes	[126,141,142]
	Gynica Cannabinoids	Early clinical	Human	Clinical symptom relief under investigation	[126]

↓: Reduced.

**Table 3:** The renin–angiotensin system-associated studies.

Study/Review	Year	Model/ Population	Main Findings	References
Expression of Angiotensin II Types 1 and 2 Receptors in Endometriosis	2016	Premenopausal Japanese women (endometriosis vs. controls)	Both AT1 and AT2 receptors are expressed in endometrial tissue of women with and without endometriosis; expression patterns may differ between groups.	[185]
Expression of RAS genes in endometrial samples	2017	Cancerous and non-cancerous endometrial samples	AGT, AGTR1, ACE1, and ACE2 genes were detected in most endometrial samples, indicating broad RAS component expressions in endometrial tissue.	[186]
The role of the renin–angiotensin system in regulating endometrial angiogenesis	2020	Human endometrial tissue: literature review and pilot study	AT1R and AT2R (angiotensin II receptors) are expressed in all endometrial compartments; altered expression is linked to endometrial vascular changes. RAS may influence angiogenesis relevant to endometriosis and implantation.	[187]
Role of Renin-Angiotensin-Aldosterone System and Cortisol in Endometriosis	2022	Women with endometriosis vs. healthy controls	Women with endometriosis had significantly lower plasma renin and higher aldosterone-renin ratio (ARR); systolic blood pressure was higher. Suggests altered RAS activity in endometriosis.	[188]
Physiological and pathological roles of Ang II and Ang-(1–7) in the female reproductive system	2022	Review (human and animal studies)	Ang II and its receptors are active in the female reproductive tract, including the endometrium; involved in vascular and inflammatory processes relevant to endometriosis.	[181]

## Data Availability

All data are provided in the review.

## References

[1] World Health Organization Endometriosis. https://www.who.int/news-room/fact-sheets/detail/endometriosis.

[2] Gete D.G., Doust J., Mortlock S., Montgomery G., Mishra G.D. (2023). Impact of Endometriosis on Women’s Health-Related Quality of Life: A National Prospective Cohort Study. Maturitas.

[3] Mayo Clinic Endometriosis. n.d. https://www.mayoclinic.org/diseases-conditions/endometriosis/symptoms-causes/syc-20354656.

[4] Menni K., Facchetti L., Cabassa P. (2016). Extragenital Endometriosis: Assessment with MR Imaging. A Pictorial Review. Br. J. Radiol..

[5] Nezhat C.R., Oskotsky T.T., Robinson J.F., Fisher S.J., Tsuei A., Liu B., Irwin J.C., Gaudilliere B., Sirota M., Stevenson D.K. (2025). Real World Perspectives on Endometriosis Disease Phenotyping through Surgery, Omics, Health Data, and Artificial Intelligence. npj Womens Health.

[6] Tosti C., Pinzauti S., Santulli P., Chapron C., Petraglia F. (2015). Pathogenetic Mechanisms of Deep Infiltrating Endometriosis. Reprod. Sci..

[7] Lee S.-Y., Koo Y.-J., Lee D.-H. (2021). Classification of Endometriosis. Yeungnam Univ. J. Med..

[8] Carneiro M.M. (2025). Weighing up GnRH Agonist Therapy for Endometriosis: Outcomes and the Treatment Paradigm. Expert Opin. Pharmacother..

[9] Wang H., Gan Z., Wang Y., Hu D., Zhang L., Ye F., Duan P. (2025). A Noninvasive Menstrual Blood-Based Diagnostic Platform for Endometriosis Using Digital Droplet Enzyme-Linked Immunosorbent Assay and Single-Cell RNA Sequencing. Research.

[10] Harder C., Velho R.V., Brandes I., Sehouli J., Mechsner S. (2024). Assessing the True Prevalence of Endometriosis: A Narrative Review of Literature Data. Int. J. Gynecol. Obstet..

[11] Benlioglu C., Telek S.B., Ata B. (2025). Reproductive Outcomes in Infertile Women with Endometriosis Undergoing ART. Gynecol. Obstet. Investig..

[12] Moen M.H., Magnus P. (1993). The Familial Risk of Endometriosis. Acta Obstet. Gynecol. Scand..

[13] Guare L.A., Das J., Caruth L., Rajagopalan A., Akerele A.T., Brumpton B.M., Chen T.-T., Kottyan L., Lin Y.-F., Moreno E. (2024). Expanding the Genetic Landscape of Endometriosis: Integrative-Omics Analyses Uncover Key Pathways from a Multi-Ancestry Study of over 900,000 Women. medRxiv.

[14] Liu Y., Liu Y., Li W., Sun X. (2024). The Association between Genetically Predicted Systemic Inflammatory Regulators and Endometriosis: A Bidirectional Mendelian Randomization Study. Medicine.

[15] Nnoaham K.E., Webster P., Kumbang J., Kennedy S.H., Zondervan K.T. (2012). Is Early Age at Menarche a Risk Factor for Endometriosis? A Systematic Review and Meta-Analysis of Case-Control Studies. Fertil. Steril..

[16] Wei M., Cheng Y., Bu H., Zhao Y., Zhao W. (2016). Length of Menstrual Cycle and Risk of Endometriosis: A Meta-Analysis of 11 Case–Control Studies. Medicine.

[17] Darrow S.L., Vena J.E., Batt R.E., Zielezny M.A., Michalek A.M., Selman S. (1993). Menstrual Cycle Characteristics and the Risk of Endometriosis. Epidemiology.

[18] Treloar S.A., Bell T.A., Nagle C.M., Purdie D.M., Green A.C. (2010). Early Menstrual Characteristics Associated with Subsequent Diagnosis of Endometriosis. Am. J. Obstet. Gynecol..

[19] El-Hadad S., Lässer D., Sachs M.-K., Schwartz A.S.K., Haeberlin F., Von Orelli S., Eberhard M., Leeners B. (2023). Dysmenorrhea in Adolescents Requires Careful Investigation of Endometriosis—An Analysis of Early Menstrual Experiences in a Large Case-Control Study. Front. Reprod. Health.

[20] Nicolaus K., Bräuer D., Sczesny R., Lehmann T., Diebolder H., Runnebaum I.B. (2019). Unexpected Coexistent Endometriosis in Women with Symptomatic Uterine Leiomyomas Is Independently Associated with Infertility, Nulliparity and Minor Myoma Size. Arch. Gynecol. Obstet..

[21] Parazzini F., Esposito G., Tozzi L., Noli S., Bianchi S. (2017). Epidemiology of Endometriosis and Its Comorbidities. Eur. J. Obstet. Gynecol. Reprod. Biol..

[22] Gupta S., Harlev A., Agarwal A., Pandithurai E. (2015). Predisposing and Protective Factors of Endometriosis. Endometriosis.

[23] Bulun S.E. (2022). Endometriosis Caused by Retrograde Menstruation: Now Demonstrated by DNA Evidence. Fertil. Steril..

[24] D’Hooghe T.M. (2002). Endometriosis, Retrograde Menstruation and Peritoneal Inflammation in Women and in Baboons. Hum. Reprod. Update.

[25] Sampson J.A. (1927). Peritoneal Endometriosis Due to the Menstrual Dissemination of Endometrial Tissue into the Peritoneal Cavity. Am. J. Obstet. Gynecol..

[26] Vercellini P., Salmeri N., Somigliana E., Piccini M., Caprara F., Viganò P., De Matteis S. (2024). Müllerian Anomalies and Endometriosis as Potential Explanatory Models for the Retrograde Menstruation/Implantation and the Embryonic Remnants/Celomic Metaplasia Pathogenic Theories: A Systematic Review and Meta-Analysis. Hum. Reprod..

[27] Pitot M.A., Bookwalter C.A., Dudiak K.M. (2020). Müllerian Duct Anomalies Coincident with Endometriosis: A Review. Abdom. Radiol..

[28] Lamceva J., Uljanovs R., Strumfa I. (2023). The Main Theories on the Pathogenesis of Endometriosis. Int. J. Mol. Sci..

[29] Shah D.K., Correia K.F., Vitonis A.F., Missmer S.A. (2013). Body Size and Endometriosis: Results from 20 Years of Follow-up within the Nurses’ Health Study II Prospective Cohort. Hum. Reprod..

[30] Liu Y., Zhang W. (2017). Association between Body Mass Index and Endometriosis Risk: A Meta-Analysis. Oncotarget.

[31] Lafay Pillet M.-C., Schneider A., Borghese B., Santulli P., Souza C., Streuli I., De Ziegler D., Chapron C. (2012). Deep Infiltrating Endometriosis Is Associated with Markedly Lower Body Mass Index: A 476 Case-Control Study. Hum. Reprod..

[32] Rowlands I.J., Hockey R., Abbott J.A., Montgomery G.W., Mishra G.D. (2022). Body Mass Index and the Diagnosis of Endometriosis: Findings from a National Data Linkage Cohort Study. Obes. Res. Clin. Pract..

[33] Giudice L.C. (2010). Endometriosis. N. Engl. J. Med..

[34] Vannuccini S., Clemenza S., Rossi M., Petraglia F. (2022). Hormonal Treatments for Endometriosis: The Endocrine Background. Rev. Endocr. Metab. Disord..

[35] Da Costa K.D.A., Malvezzi H., Dobo C., Neme R.M., Filippi R.Z., Aloia T.P.A., Prado E.R., Meola J., Piccinato C.D.A. (2022). Site-Specific Regulation of Sulfatase and Aromatase Pathways for Estrogen Production in Endometriosis. Front. Mol. Biosci..

[36] Matsuzaki S., Canis M., Pouly J.-L., Déchelotte P.J., Mage G. (2006). Analysis of Aromatase and 17β-Hydroxysteroid Dehydrogenase Type 2 Messenger Ribonucleic Acid Expression in Deep Endometriosis and Eutopic Endometrium Using Laser Capture Microdissection. Fertil. Steril..

[37] Ishikawa H., Shozu M., Harada T. (2014). Aromatase Expression in Endometriosis and Its Significance. Endometriosis.

[38] Chantalat E., Valera M.-C., Vaysse C., Noirrit E., Rusidze M., Weyl A., Vergriete K., Buscail E., Lluel P., Fontaine C. (2020). Estrogen Receptors and Endometriosis. Int. J. Mol. Sci..

[39] Bulun S., Monsavais D., Pavone M., Dyson M., Xue Q., Attar E., Tokunaga H., Su E. (2012). Role of Estrogen Receptor-β in Endometriosis. Semin. Reprod. Med..

[40] Jeng C.-J., Chuang L., Shen J. (2014). A Comparison of Progestogens or Oral Contraceptives and Gonadotropin-Releasing Hormone Agonists for the Treatment of Endometriosis: A Systematic Review. Expert Opin. Pharmacother..

[41] Rojek K., Juda A., Kamińska M., Strzoda A., Strzoda A., Sowiński W., Zdybel M., Strzoda A. (2023). Progestins and Combined Oral Contraceptives in the Hormonal Treatment of Endometriosis—A Review. Eur. J. Clin. Exp. Med..

[42] Kauppila A. (1998). Reappraisal of Progestins in Endometriosis Therapy. Eur. J. Endocrinol..

[43] Schweppe K.-W. (2009). The Place of Dydrogesterone in the Treatment of Endometriosis and Adenomyosis. Maturitas.

[44] Oosterlynck D.J., Meuleman C., Waer M., Vandeputte M., Koninckx P.R. (1992). The Natural Killer Activity of Peritoneal Fluid Lymphocytes Is Decreased in Women with Endometriosis. Fertil. Steril..

[45] Lebovic D.I., Mueller M.D., Taylor R.N. (2001). Immunobiology of Endometriosis. Fertil. Steril..

[46] Gazvani R., Templeton A. (2002). Peritoneal Environment, Cytokines and Angiogenesis in the Pathophysiology of Endometriosis. Reproduction.

[47] Harada T., Iwabe T., Terakawa N. (2001). Role of Cytokines in Endometriosis. Fertil. Steril..

[48] Xia T., Zeng K., Peng Q., Wu X., Lei X. (2023). Clinical Significance of Serum Th1/Th2 Cytokines in Patients with Endometriosis. Women Health.

[49] Abramiuk M., Grywalska E., Małkowska P., Sierawska O., Hrynkiewicz R., Niedźwiedzka-Rystwej P. (2022). The Role of the Immune System in the Development of Endometriosis. Cells.

[50] Le N.X.H., Loret De Mola J.R., Bremer P., Groesch K., Wilson T., Diaz-Sylvester P., Braundmeier-Fleming A.G. (2021). Alteration of Systemic and Uterine Endometrial Immune Populations in Patients with Endometriosis. Am. J. Rep. Immunol..

[51] Sisnett D.J., Zutautas K.B., Miller J.E., Lingegowda H., Ahn S.H., McCallion A., Bougie O., Lessey B.A., Tayade C. (2024). The Dysregulated IL-23/TH17 Axis in Endometriosis Pathophysiology. J. Immunol..

[52] Berbic M., Fraser I.S. (2011). Regulatory T Cells and Other Leukocytes in the Pathogenesis of Endometriosis. J. Reprod. Immunol..

[53] Olkowska-Truchanowicz J., Bocian K., Maksym R.B., Bialoszewska A., Wlodarczyk D., Baranowski W., Zabek J., Korczak-Kowalska G., Malejczyk J. (2013). CD4+ CD25+ FOXP3+ Regulatory T Cells in Peripheral Blood and Peritoneal Fluid of Patients with Endometriosis. Hum. Reprod..

[54] Khan K.N., Yamamoto K., Fujishita A., Muto H., Koshiba A., Kuroboshi H., Saito S., Teramukai S., Nakashima M., Kitawaki J. (2019). Differential Levels of Regulatory T Cells and T-Helper-17 Cells in Women With Early and Advanced Endometriosis. J. Clin. Endocrinol. Metab..

[55] Shigesi N., Kvaskoff M., Kirtley S., Feng Q., Fang H., Knight J.C., Missmer S.A., Rahmioglu N., Zondervan K.T., Becker C.M. (2019). The Association between Endometriosis and Autoimmune Diseases: A Systematic Review and Meta-Analysis. Hum. Reprod. Update.

[56] Sinaii N. (2002). High Rates of Autoimmune and Endocrine Disorders, Fibromyalgia, Chronic Fatigue Syndrome and Atopic Diseases among Women with Endometriosis: A Survey Analysis. Hum. Reprod..

[57] Diamanti-Kandarakis E., Bourguignon J.-P., Giudice L.C., Hauser R., Prins G.S., Soto A.M., Zoeller R.T., Gore A.C. (2009). Endocrine-Disrupting Chemicals: An Endocrine Society Scientific Statement. Endocr. Rev..

[58] Heindel J.J., Blumberg B., Cave M., Machtinger R., Mantovani A., Mendez M.A., Nadal A., Palanza P., Panzica G., Sargis R. (2017). Metabolism Disrupting Chemicals and Metabolic Disorders. Reprod. Toxicol..

[59] Rudel R.A., Attfield K.R., Schifano J.N., Brody J.G. (2007). Chemicals Causing Mammary Gland Tumors in Animals Signal New Directions for Epidemiology, Chemicals Testing, and Risk Assessment for Breast Cancer Prevention. Cancer.

[60] Mustieles V., Pérez-Lobato R., Olea N., Fernández M.F. (2015). Bisphenol A: Human Exposure and Neurobehavior. NeuroToxicology.

[61] Vercellini P., Viganò P., Somigliana E., Fedele L. (2014). Endometriosis: Pathogenesis and Treatment. Nat. Rev. Endocrinol..

[62] Dunselman G.A.J., Vermeulen N., Becker C., Calhaz-Jorge C., D’Hooghe T., De Bie B., Heikinheimo O., Horne A.W., Kiesel L., Nap A. (2014). ESHRE Guideline: Management of Women with Endometriosis. Hum. Reprod..

[63] Bender Atik R., Christiansen O.B., Elson J., Kolte A.M., Lewis S., Middeldorp S., Mcheik S., Peramo B., Quenby S., The ESHRE Guideline Group on RPL (2022). ESHRE Guideline: Recurrent Pregnancy Loss: An Update in 2022. Hum. Reprod. Open.

[64] The Practice Committee of the American Society for Reproductive Medicine (2012). Endometriosis and Infertility: A Committee Opinion. Fertil. Steril..

[65] Armour M., Sinclair J., Chalmers K.J., Smith C.A. (2019). Self-Management Strategies amongst Australian Women with Endometriosis: A National Online Survey. BMC Complement. Altern. Med..

[66] Koga K., Osuga Y., Yoshino O., Hirota Y., Yano T., Tsutsumi O., Taketani Y. (2005). Elevated Interleukin-16 Levels in the Peritoneal Fluid of Women with Endometriosis May Be a Mechanism for Inflammatory Reactions Associated with Endometriosis. Fertil. Steril..

[67] Kuteken F.S., Lucia C., Lancellotti P. (2012). Expressão de Mediadores Neurotróficos e Pró-Inflamatórios Na Endometriose de Reto e Sigmoide Expression of Neurotrophic and Inflammatory Mediators in Rectosigmoid Endometriosis. Rev. Bras. Ginecol. Obstet..

[68] Scholl B., Bersinger N.A., Kuhn A., Mueller M.D. (2009). Correlation between Symptoms of Pain and Peritoneal Fluid Inflammatory Cytokine Concentrations in Endometriosis. Gynecol. Endocrinol..

[69] Riley C.F., Moen M.H., Videm V. (2007). Inflammatory Markers in Endometriosis: Reduced Peritoneal Neutrophil Response in Minimal Endometriosis. Acta Obstet. Gynecol. Scand..

[70] Capellino S., Montagna P., Villaggio B., Sulli A., Soldano S., Ferrero S., Remorgida V., Cutolo M. (2006). Role of Estrogens in Inflammatory Response: Expression of Estrogen Receptors in Peritoneal Fluid Macrophages from Endometriosis. Ann. N. Y. Acad. Sci..

[71] Montagna P., Capellino S., Villaggio B., Remorgida V., Ragni N., Cutolo M., Ferrero S. (2008). Peritoneal Fluid Macrophages in Endometriosis: Correlation between the Expression of Estrogen Receptors and Inflammation. Fertil. Steril..

[72] Liu Z. (2016). Inflammation and Endometriosis. Front. Biosci..

[73] Vallvé-Juanico J., Houshdaran S., Giudice L.C. (2019). The Endometrial Immune Environment of Women with Endometriosis. Hum. Reprod. Update.

[74] Xiao F., Liu X., Guo S.-W. (2022). Interleukin-33 Derived from Endometriotic Lesions Promotes Fibrogenesis through Inducing the Production of Profibrotic Cytokines by Regulatory T Cells. Biomedicines.

[75] Wu X.-G., Chen J.-J., Zhou H.-L., Wu Y., Lin F., Shi J., Wu H.-Z., Xiao H.-Q., Wang W. (2021). Identification and Validation of the Signatures of Infiltrating Immune Cells in the Eutopic Endometrium Endometria of Women With Endometriosis. Front. Immunol..

[76] Fan D., Wang X., Shi Z., Jiang Y., Zheng B., Xu L., Zhou S. (2023). Understanding Endometriosis from an Immunomicroenvironmental Perspective. Chin. Med. J..

[77] Tanaka Y., Mori T., Ito F., Koshiba A., Takaoka O., Kataoka H., Maeda E., Okimura H., Mori T., Kitawaki J. (2017). Exacerbation of Endometriosis Due To Regulatory T-Cell Dysfunction. J. Clin. Endocrinol. Metab..

[78] Bulun S.E., Nezhat C. (2016). Aromatase, microRNA, and Inflammation: A Complex Relationship. Fertil. Steril..

[79] Lopez A., Cruz M.L., Chompre G., Hernández S., Isidro R.A., Flores I., Appleyard C.B. (2020). Influence of Stress on the Vitamin D-Vitamin D Receptor System, Macrophages, and the Local Inflammatory Milieu in Endometriosis. Reprod. Sci..

[80] Gołąbek-Grenda A., Juzwa W., Kaczmarek M., Olejnik A. (2024). Resveratrol and Its Natural Analogs Mitigate Immune Dysregulation and Oxidative Imbalance in the Endometriosis Niche Simulated in a Co-Culture System of Endometriotic Cells and Macrophages. Nutrients.

[81] Jiang T., Chen Y., Gu X., Miao M., Hu D., Zhou H., Chen J., Teichmann A.T., Yang Y. (2023). Review of the Potential Therapeutic Effects and Molecular Mechanisms of Resveratrol on Endometriosis. Int. J. Womens Health.

[82] Mikuš M., Vitale S.G., Ćorić M., Zajec V., Ciebiera M., Carugno J., D’alterio M.N., Herman M., Puževski T., Angioni S. (2022). State of the Art, New Treatment Strategies, and Emerging Drugs for Non-Hormonal Treatment of Endometriosis: A Systematic Review of Randomized Control Trials. Gynecol. Endocrinol..

[83] Dolmans M.-M., Donnez J. (2022). Emerging Drug Targets for Endometriosis. Biomolecules.

[84] Sanamiri K., Mahdian S., Moini A., Shahhoseini M. (2024). Non-Hormonal Therapy for Endometriosis Based on Angiogenesis Oxidative Stress and Inflammation. Int. J. Fertil. Steril..

[85] Maybin J.A., Critchley H.O.D., Jabbour H.N. (2011). Inflammatory Pathways in Endometrial Disorders. Mol. Cell. Endocrinol..

[86] Thiruchelvam U., Wingfield M., O’Farrelly C. (2015). Natural Killer Cells: Key Players in Endometriosis. Am. J. Rep. Immunol..

[87] Reis J.L., Rosa N.N., Ângelo-Dias M., Martins C., Borrego L.M., Lima J. (2022). Natural Killer Cell Receptors and Endometriosis: A Systematic Review. Int. J. Mol. Sci..

[88] Yang Y.-M., Yang W.-X. (2017). Epithelial-to-Mesenchymal Transition in the Development of Endometriosis. Oncotarget.

[89] Zhang Q., Duan J., Olson M., Fazleabas A., Guo S.-W. (2016). Cellular Changes Consistent With Epithelial–Mesenchymal Transition and Fibroblast-to-Myofibroblast Transdifferentiation in the Progression of Experimental Endometriosis in Baboons. Reprod. Sci..

[90] Shi L.B., Zhou F., Zhu H.Y., Huang D., Jin X.Y., Li C., Dai Y., Pan Y.B., Zhang S.Y. (2017). Transforming Growth Factor Beta1 from Endometriomas Promotes Fibrosis in Surrounding Ovarian Tissues via Smad2/3 Signaling†. Biol. Reprod..

[91] McLaren J. (2000). Vascular Endothelial Growth Factor and Endometriotic Angiogenesis. Hum. Reprod. Update.

[92] McLaren J., Prentice A., Charnock-Jones D.S., Millican S.A., Müller K.H., Sharkey A.M., Smith S.K. (1996). Vascular Endothelial Growth Factor Is Produced by Peritoneal Fluid Macrophages in Endometriosis and Is Regulated by Ovarian Steroids. J. Clin. Investig..

[93] Kianpour M., Nematbakhsh M., Ahmadi S.M., Jafarzadeh M., Hajjarian M., Pezeshki Z., Safari T., Eshraghi-Jazi F. (2013). Serum and Peritoneal Fluid Levels of Vascular Endothelial Growth Factor in Women with Endometriosis. Int. J. Fertil. Steril..

[94] Chung M.S., Han S.J. (2022). Endometriosis-Associated Angiogenesis and Anti-Angiogenic Therapy for Endometriosis. Front. Glob. Womens Health.

[95] Malvezzi H., Cestari B.A., Meola J., Podgaec S. (2023). Higher Oxidative Stress in Endometriotic Lesions Upregulates Senescence-Associated P16ink4a and β-Galactosidase in Stromal Cells. Int. J. Mol. Sci..

[96] Cho Y.J., Kim H.Y. (2018). Oxidative Stress and Endometriosis. Kosin Med. J..

[97] Clower L., Fleshman T., Geldenhuys W.J., Santanam N. (2022). Targeting Oxidative Stress Involved in Endometriosis and Its Pain. Biomolecules.

[98] Surya Udayana I.G.N.B., Praja Adnyana I.B.P., Diningrat M.A., Setiawan W.A. (2022). Association of Endometriosis and Oxidative Stress. Eur. J. Med. Health Sci..

[99] Daud V.P.A., Dyarma K.R., Farida L.S. (2024). Antioxidant Agent to Improve Endometriosis Related Pain (Dysmenorrhea, Dyspareunia, Pelvic Pain): A Systematic Review and Meta-Analysis. Asian J. Health Res..

[100] Akhtar M.N., Saeed R., Saeed F., Asghar A., Ghani S., Ateeq H., Ahmed A., Rasheed A., Afzaal M., Waheed M. (2023). Silymarin: A Review on Paving the Way towards Promising Pharmacological Agent. Int. J. Food Prop..

[101] Mirzaei N., Sadatmahalleh S.J., Rouholamin S., Nasiri M. (2022). Effect of Silymarin on Interleukin-6 Level and Size of Endometrioma Lesions in Women with Ovarian Endometriosis: A Randomized, Double-Blind Placebo-Controlled Trial. Res. Sq..

[102] Anastasi E., Scaramuzzino S., Viscardi M.F., Viggiani V., Piccioni M.G., Cacciamani L., Merlino L., Angeloni A., Muzii L., Porpora M.G. (2023). Efficacy of N-Acetylcysteine on Endometriosis-Related Pain, Size Reduction of Ovarian Endometriomas, and Fertility Outcomes. Int. J. Environ. Res. Public Health.

[103] Porpora M.G., Brunelli R., Costa G., Imperiale L., Krasnowska E.K., Lundeberg T., Nofroni I., Piccioni M.G., Pittaluga E., Ticino A. (2013). A Promise in the Treatment of Endometriosis: An Observational Cohort Study on Ovarian Endometrioma Reduction by N-Acetylcysteine. Evid. Based Complement. Altern. Med..

[104] Harzif A.K., Ummah N., Puspawardani A.R., Nurbaeti P., Wiweko B. (2024). Effect of Omega-3 Fatty Acids on Endometriosis: A Systematic Review. Bali Med. J..

[105] Abokhrais I.M., Denison F.C., Whitaker L.H.R., Saunders P.T.K., Doust A., Williams L.J., Horne A.W. (2020). A Two-Arm Parallel Double-Blind Randomised Controlled Pilot Trial of the Efficacy of Omega-3 Polyunsaturated Fatty Acids for the Treatment of Women with Endometriosis-Associated Pain (PurFECT1). PLoS ONE.

[106] Vallée A., Lecarpentier Y. (2020). Curcumin and Endometriosis. Int. J. Mol. Sci..

[107] Gudarzi R., Shabani F., Mohammad-Alizadeh-Charandabi S., Naghshineh E., Shaseb E., Mirghafourvand M. (2024). Effect of Curcumin on Painful Symptoms of Endometriosis: A Triple-blind Randomized Controlled Trial. Phytother. Res..

[108] Sapkota Y., Steinthorsdottir V., Morris A.P., Fassbender A., Rahmioglu N., De Vivo I., Buring J.E., Zhang F., Edwards T.L., Jones S. (2017). Meta-Analysis Identifies Five Novel Loci Associated with Endometriosis Highlighting Key Genes Involved in Hormone Metabolism. Nat. Commun..

[109] Painter J.N., Anderson C.A., Nyholt D.R., Macgregor S., Lin J., Lee S.H., Lambert A., Zhao Z.Z., Roseman F., Guo Q. (2011). Genome-Wide Association Study Identifies a Locus at 7p15.2 Associated with Endometriosis. Nat. Genet..

[110] Guo S.-W. (2009). Epigenetics of Endometriosis. Mol. Hum. Reprod..

[111] Méar L., Herr M., Fauconnier A., Pineau C., Vialard F. (2020). Polymorphisms and Endometriosis: A Systematic Review and Meta-Analyses. Hum. Reprod. Update.

[112] Akbar A. (2025). In Silico Analysis of Non-Coding RNA Regulation in Human Gene Expression: A Systematic Computational Approach to Understanding Regulatory Networks. bioRxiv.

[113] Zhang T. (2025). Non-Coding RNAs in Tumor Biology: Exploring miRNAs, lncRNAs, and circRNAs Roles and Therapeutic Potentials. Theor. Nat. Sci..

[114] Yuad H., Utama B.I., Lipoeto N.I., Putra A.E. (2025). Gut Microbiota Profile in Endometriosis Patients at Dr. M. Djamil Padang, West Sumatra, Indonesia. J. OBGIN EMAS.

[115] Verma S., Sowdhamini R. (2025). Toll-like Receptor 4 Pathway Evolutionary Trajectory and Functional Emergence. Front. Immunol..

[116] Meria S.Z., Yuad H., Putra A.E. (2025). Comparison of Vaginal Microbiota Profiles in Patients With Endometriosis and Without Endometriosis. Andalas Obstet. Gynecol. J..

[117] Horne A.W., Ahmad S.F., Carter R., Simitsidellis I., Greaves E., Hogg C., Morton N.M., Saunders P.T.K. (2019). Repurposing Dichloroacetate for the Treatment of Women with Endometriosis. Proc. Natl. Acad. Sci. USA.

[118] Chiara F., Allegra S., Caudana M., Mula J., Turco D., Liuzzi S., Puccinelli M.P., Mengozzi G., De Francia S. (2025). Central Serous Chorioretinopathy in Endometriosis Treatment with Progestogen: A Metabolic Understanding. Life.

[119] Zhou L., Liu L., Chai W., Zhao T., Jin X., Guo X., Han L., Yuan C. (2019). Dichloroacetic Acid Upregulates Apoptosis of Ovarian Cancer Cells by Regulating Mitochondrial Function. OncoTargets Ther..

[120] Alkarakooly Z., Al-Anbaky Q.A., Kannan K., Ali N. (2018). Metabolic Reprogramming by Dichloroacetic Acid Potentiates Photodynamic Therapy of Human Breast Adenocarcinoma MCF-7 Cells. PLoS ONE.

[121] Pan J.G., Mak T.W. (2007). Metabolic Targeting as an Anticancer Strategy: Dawn of a New Era?. Sci. STKE.

[122] Harvey M.E., Shi M., Oh Y., Mitchell D.A., Slayden O.D., MacLean J.A., Hayashi K. (2025). Multiple Lesion Inductions Intensify Central Sensitization Driven by Neuroinflammation in a Mouse Model of Endometriosis. bioRxiv.

[123] Scarpina F., Navarra M.E., Varallo G., Bernorio R. (2025). The Role of Interoceptive Sensibility on Central Sensitization to Pain in Vulvodynia. J. Sex. Med..

[124] Whitaker L.H.R., Page C., Morgan C., Horne A.W., Saunders P.T.K. (2024). Endometriosis: Cannabidiol Therapy for Symptom Relief. Trends Pharmacol. Sci..

[125] Thalia O.P. (2024). Immunomodulation via the Endocannabinoid System: Mechanisms and Therapeutic Potentials. Res. Invent. J. Biol. Appl. Sci..

[126] Filho R.F.B., Marchiori C.H., Malheiros K.D.P. (2024). Relationship of the Endocannabinoid System with Hormonal Functions. Middle East Res. J. Med. Sci..

[127] Kimber-Trojnar Ż., Dłuski D.F., Wierzchowska-Opoka M., Ruszała M., Leszczyńska-Gorzelak B. (2022). Metformin as a Potential Treatment Option for Endometriosis. Cancers.

[128] Yilmaz B., Sucak A., Kilic S., Aksakal O., Aksoy Y., Lortlar N., Sut N., Gungor T. (2010). Metformin Regresses Endometriotic Implants in Rats by Improving Implant Levels of Superoxide Dismutase, Vascular Endothelial Growth Factor, Tissue Inhibitor of Metalloproteinase-2, and Matrix Metalloproteinase-9. Am. J. Obstet. Gynecol..

[129] Lasker N., Banu J., Munira S.M., Al Tarique M.M., Anwary S.A., Ghosh T., Sultana S., Laila R., Akter A. (2024). Effects of Pentoxifylline and Metformin Combination Therapy Compared to Metformin Alone in Infertile Women with Symptomatic Endometrioma. Int. J. Reprod. Contracept. Obstet. Gynecol..

[130] A.Omer N., A.Taher M., Dh Skheel Aljebory H. (2017). Effect of Metformin Treatment on Some Blood Biomarkers in Women with Endometriosis. Iraqi J. Pharm. Sci..

[131] Takemura Y., Osuga Y., Yoshino O., Hasegawa A., Hirata T., Hirota Y., Nose E., Morimoto C., Harada M., Koga K. (2007). Metformin Suppresses Interleukin (IL)-1β-Induced IL-8 Production, Aromatase Activation, and Proliferation of Endometriotic Stromal Cells. J. Clin. Endocrinol. Metab..

[132] Neto Cerqueira A.C., Botelho M., Rodrigues A., Lamas S., Araújo B., Guimarães J.T., Gouveia A., Almeida H., Neves D. (2024). P-331 Unlocking Metformin’s Potential: Exploring Its Impact on Inflammation and Oxidative Pathways in an Endometriosis Mouse Model. Hum. Reprod..

[133] Cheng J., Li C., Ying Y., Lv J., Qu X., McGowan E., Lin Y., Zhu X. (2022). Metformin Alleviates Endometriosis and Potentiates Endometrial Receptivity via Decreasing VEGF and MMP9 and Increasing Leukemia Inhibitor Factor and HOXA10. Front. Pharmacol..

[134] Mulyantoro I., Indrapraja O., Widjiati W., Noerpramana N.P. (2020). Effect of Metformin on Bcl-2/Bax Expression Ratio and Endometrial Implants: A Mouse Model in Endometriosis Study. J. Biomed. Transl. Res..

[135] Shao X., Li C., Liang J., Changzhong L. (2024). Metformin Enhances Epithelial Cell Growth Inhibition via the Protein Kinase–Insulin-like Growth Factor Binding Protein-1 Pathway. J. Obstet. Gynaecol..

[136] Xu J.-N., Zeng C., Zhou Y., Peng C., Zhou Y.-F., Xue Q. (2014). Metformin Inhibits *StAR* Expression in Human Endometriotic Stromal Cells via AMPK-Mediated Disruption of CREB-CRTC2 Complex Formation. J. Clin. Endocrinol. Metab..

[137] Zhou Y., Xu J.-N., Zeng C., Li X., Zhou Y.-F., Qi Y., Xue Q. (2015). Metformin Suppresses Prostaglandin E2-Induced Cytochrome P450 Aromatase Gene Expression and Activity via Stimulation of AMP-Activated Protein Kinase in Human Endometriotic Stromal Cells. Reprod. Sci..

[138] Shoshan Q., Elshamy M., Wageh A., Thabet M. (2024). Cabergoline, Metformin and Clomiphene Citrate Therapy in Infertile Female with Mild Endometriosis: A Randomized Clinical Trial. Egypt. J. Fertil. Steril..

[139] Stochino-Loi E., Major A.L., Gillon T.E.R., Ayoubi J.-M., Feki A., Bouquet De Joliniere J. (2021). Metformin, the Rise of a New Medical Therapy for Endometriosis? A Systematic Review of the Literature. Front. Med..

[140] Brown J., Crawford T.J., Allen C., Hopewell S., Prentice A. (2017). Nonsteroidal Anti-Inflammatory Drugs for Pain in Women with Endometriosis. Cochrane Database Syst. Rev..

[141] Cabergoline in the Treatment of Endometriosis-Associated Pain. https://clinicaltrials.gov/ct2/show/NCT05231060.

[142] Pellicer N., Galliano D., Herraiz S., Bagger Y.Z., Arce J.-C., Pellicer A. (2021). Use of Dopamine Agonists to Target Angiogenesis in Women with Endometriosis. Hum. Reprod..

[143] Novella-Maestre E., Carda C., Ruiz-Sauri A., Garcia-Velasco J.A., Simon C., Pellicer A. (2010). Identification and Quantification of Dopamine Receptor 2 in Human Eutopic and Ectopic Endometrium: A Novel Molecular Target for Endometriosis Therapy1. Biol. Reprod..

[144] DiVasta A.D., Stamoulis C., Gallagher J.S., Laufer M.R., Anchan R., Hornstein M.D. (2021). Nonhormonal Therapy for Endometriosis: A Randomized, Placebo-Controlled, Pilot Study of Cabergoline versus Norethindrone Acetate. F S Rep..

[145] The 7 Hottest Biotech Companies Advancing Endometriosis Care. https://www.labiotech.eu/best-biotech/biotech-companies-endometriosis-therapies/.

[146] Groundbreaking Immunotherapy to Target Endometriosis. https://www.femtechworld.co.uk/pain-conditions/groundbreaking-immunotherapy-to-target-endometriosis//.

[147] Madasu C., Liao Z., Parks S.E., Sharma K.L., Bohren K.M., Ye Q., Li F., Palaniappan M., Tan Z., Yuan F. (2024). Identification of Potent Pan-Ephrin Receptor Kinase Inhibitors Using DNA-Encoded Chemistry Technology. Proc. Natl. Acad. Sci. USA.

[148] Murakami M., Sato H., Taketomi Y. (2020). Updating Phospholipase A2 Biology. Biomolecules.

[149] Perelló M., González-Foruria I., Castillo P., Martínez-Florensa M., Lozano F., Balasch J., Carmona F. (2017). Oral Administration of Pentoxifylline Reduces Endometriosis-Like Lesions in a Nude Mouse Model. Reprod. Sci..

[150] Grammatis A.L., Georgiou E.X., Becker C.M. (2021). Pentoxifylline for the Treatment of Endometriosis-Associated Pain and Infertility. Cochrane Database Syst. Rev..

[151] Koninckx P.R., Craessaerts M., Timmerman D., Cornillie F., Kennedy S. (2008). Anti-TNF- Treatment for Deep Endometriosis-Associated Pain: A Randomized Placebo-Controlled Trial. Hum. Reprod..

[152] Lu D., Song H., Shi G., The Cochrane Collaboration (2010). Anti-TNF-α Treatment for Pelvic Pain Associated with Endometriosis. Cochrane Database of Systematic Reviews.

[153] Domiciano C.B., Costa Filho A., Camilo Neto G., Felipe D.H.D.C.N., Maia A.C., Oliveira D.C.N.D., Cavalcante B.V.B., Ferreira P.C., Pereira A.L.G.X.D.N. (2022). O Impacto Do Tratamento Com Antibióticos No Prognóstico Obstétrico de Pacientes Com Endometriose Crônica. Res. Soc. Dev..

[154] Liu J., Liu Z.A., Liu Y., Cheng L., Yan L. (2022). Impact of Antibiotic Treatment for Chronic Endometritis on Pregnancy Outcomes in Women with Reproductive Failures (RIF and RPL): A Systematic Review and Meta-Analysis. Front. Med..

[155] Luncan M., Huniadi A., Bimbo-Szuhai E., Botea M., Zaha I., Stefan L., Beiusanu C., Romanescu D., Pallag A., Bodog A. (2022). The Effectiveness of Intrauterine Antibiotic Infusion versus Oral Antibiotic Therapy in the Treatment of Chronic Endometritis in Patients during IVF (in Vitro Fertilization) Procedures. BMC Womens Health.

[156] Chadchan S.B., Cheng M., Parnell L.A., Yin Y., Schriefer A., Mysorekar I.U., Kommagani R. (2019). Antibiotic Therapy with Metronidazole Reduces Endometriosis Disease Progression in Mice: A Potential Role for Gut Microbiota. Hum. Reprod..

[157] Lu F., Wei J., Zhong Y., Feng Y., Ma B., Xiong Y., Wei K., Tan B., Chen T. (2022). Antibiotic Therapy and Vaginal Microbiota Transplantation Reduce Endometriosis Disease Progression in Female Mice via NF-κB Signaling Pathway. Front. Med..

[158] Cicinelli E., De Ziegler D., Vitagliano A. (2024). In Women with Endometriosis, Effective Treatment of Chronic Endometritis with Antibiotics Lowers Serum CA-125 Levels. Fertil. Steril..

[159] Salliss M.E., Farland L.V., Mahnert N.D., Herbst-Kralovetz M.M. (2021). The Role of Gut and Genital Microbiota and the Estrobolome in Endometriosis, Infertility and Chronic Pelvic Pain. Hum. Reprod. Update.

[160] (2025). Recent Advancements in Research on Endometriosis. https://blog.mdpi.com/2025/03/04/recent-advancements-in-research-on-endometriosis.

[161] Nenicu A., Korbel C., Gu Y., Menger M.D., Laschke M.W. (2014). Combined Blockade of Angiotensin II Type 1 Receptor and Activation of Peroxisome Proliferator-Activated Receptor- by Telmisartan Effectively Inhibits Vascularization and Growth of Murine Endometriosis-like Lesions. Hum. Reprod..

[162] Moazen S., Arjmand M.-H. (2024). The Role of Local Angiotensin II/Angiotensin Type 1 Receptor in Endometriosis: A Potential Target for New Treatment Approaches. Curr. Mol. Pharmacol..

[163] Zanon P., Terraciano P.B., Quandt L., Palma Kuhl C., Pandolfi Passos E., Berger M. (2024). Angiotensin II—AT1 Receptor Signalling Regulates the Plasminogen-Plasmin System in Human Stromal Endometrial Cells Increasing Extracellular Matrix Degradation, Cell Migration and Inducing a Proinflammatory Profile. Biochem. Pharmacol..

[164] Cakmak B., Cavusoglu T., Ates U., Meral A., Nacar M.C., Erbaş O. (2015). Regression of Experimental Endometriotic Implants in a Rat Model with the Angiotensin II Receptor Blocker Losartan. J. Obstet. Gynaecol. Res..

[165] Shabanian S., Khazaie M., Ferns G.A., Arjmand M.-H. (2022). Local Renin-Angiotensin System Molecular Mechanisms in Intrauterine Adhesions Formation Following Gynecological Operations, New Strategy for Novel Treatment. J. Obstet. Gynaecol..

[166] Wartan S. (2022). Pain Management Strategies and Alternative Therapies. Endometriosis.

[167] Gubbiotti M., Giannantoni A., Lamberti G., Giraudo D., Musco S. (2020). Management of the Central Nervous System Chronic Pelvic Pain. Suprapontine Lesions and Neurogenic Pelvic Dysfunctions.

[168] Simpson G., Philip M., Lucky T., Ang C., Kathurusinghe S. (2022). A Systematic Review of the Efficacy and Availability of Targeted Treatments for Central Sensitization in Women With Endometriosis. Clin. J. Pain.

[169] Zheng P., Jia S., Guo D., Chen S., Zhang W., Cheng A., Xie W., Sun G., Leng J., Lang J. (2020). Central Sensitization-Related Changes in Brain Function Activity in a Rat Endometriosis-Associated Pain Model. J. Pain Res..

[170] Quintas-Marquès L., Martínez-Zamora M.-Á., Camacho M., Gràcia M., Rius M., Ros C., Carrión A., Carmona F. (2023). Central Sensitization in Patients with Deep Endometriosis. Pain Med..

[171] Yu J., Berga S.L., Zou E., Schrepf A.D., Clauw D.J., As-Sanie S., Taylor R.N. (2023). Neurotrophins and Their Receptors, Novel Therapeutic Targets for Pelvic Pain in Endometriosis, Are Coordinately Regulated by IL-1β via the JNK Signaling Pathway. Am. J. Pathol..

[172] Rocha A.L.L., Reis F.M., Taylor R.N. (2013). Angiogenesis and Endometriosis. Obstet. Gynecol. Int..

[173] Machairiotis N., Vasilakaki S., Thomakos N. (2021). Inflammatory Mediators and Pain in Endometriosis: A Systematic Review. Biomedicines.

[174] Ruiz-Ortega M., Ruperez M., Lorenzo O., Esteban V., Blanco J., Mezzano S., Egido J. (2002). Angiotensin II Regulates the Synthesis of Proinflammatory Cytokines and Chemokines in the Kidney. Kidney Int..

[175] Casalechi M., Dela Cruz C., Lima L.C., Maciel L.P., Pereira V.M., Reis F.M. (2018). Angiotensin Peptides in the Non-Gravid Uterus: Paracrine Actions beyond Circulation. Peptides.

[176] Yu Q., Zhu D., Zou Y., Wang K., Rao P., Shen Y. (2022). Catalpol Attenuates Pulmonary Fibrosis by Inhibiting Ang II/AT1 and TGF-β/Smad-Mediated Epithelial Mesenchymal Transition. Front. Med..

[177] Valcourt U., Kowanetz M., Niimi H., Heldin C.-H., Moustakas A. (2005). TGF-β and the Smad Signaling Pathway Support Transcriptomic Reprogramming during Epithelial-Mesenchymal Cell Transition. Mol. Biol. Cell.

[178] Zhao L., Gu C., Meng Y. (2016). Meta-Analysis of the Association between Endometriosis and Polymorphisms in ACE and PAI-1. Int. J. Clin. Exp. Med..

[179] Falconer H., D’Hooghe T., Fried G. (2007). Endometriosis and Genetic Polymorphisms. Obstet. Gynecol. Surv..

[180] Ino K., Shibata K., Yamamoto E., Kajiyama H., Nawa A., Mabuchi Y., Yagi S., Minami S., Tanizaki Y., Kobayashi A. (2011). Role of the Renin-Angiotensin System in Gynecologic Cancers. Curr. Cancer Drug Targets.

[181] Liu Y., Hao H., Lan T., Jia R., Cao M., Zhou L., Zhao Z., Pan W. (2022). Physiological and Pathological Roles of Ang II and Ang- (1-7) in the Female Reproductive System. Front. Endocrinol..

[182] Herr D., Bekes I., Wulff C. (2013). Local Renin-Angiotensin System in the Reproductive System. Front. Endocrinol..

[183] Domińska K. (2020). Involvement of ACE2/Ang-(1-7)/MAS1 Axis in the Regulation of Ovarian Function in Mammals. Int. J. Mol. Sci..

[184] Shan T., Shang W., Zhang L., Zhao C., Chen W., Zhang Y., Li G. (2015). Effect of Angiotensin-(1-7) and Angiotensin II on the Proliferation and Activation of Human Endometrial Stromal Cells in Vitro. Int. J. Clin. Exp. Pathol..

[185] Nakao T., Chishima F., Sugitani M., Tsujimura R., Hayashi C., Yamamoto T. (2017). Expression of Angiotensin II Types 1 and 2 Receptors in Endometriotic Lesions. Gynecol. Obstet. Investig..

[186] Delforce S.J., Lumbers E.R., Corbisier De Meaultsart C., Wang Y., Proietto A., Otton G., Scurry J., Verrills N.M., Scott R.J., Pringle K.G. (2017). Expression of Renin–Angiotensin System (RAS) Components in Endometrial Cancer. Endocr. Connect..

[187] Qi R., Li T.C., Chen X. (2020). The Role of the Renin–Angiotensin System in Regulating Endometrial Neovascularization during the Peri-Implantation Period: Literature Review and Preliminary Data. Ther. Adv. Endocrinol..

[188] Sabbadin C., Saccardi C., Andrisani A., Vitagliano A., Marin L., Ragazzi E., Bordin L., Ambrosini G., Armanini D. (2022). Role of Renin-Angiotensin-Aldosterone System and Cortisol in Endometriosis: A Preliminary Report. Int. J. Mol. Sci..

[189] Xie Q., Zhao C., Jiang W., Li X., Ni D., Chen Y., Li X., Hua X., Shen R., Ling X. (2024). Antibiotics Improve Reproductive Outcomes after Frozen-Thaw Embryo Transfer for Chronic Endometritis Treatment, Especially in Those with Repeated Implantation Failure. BMC Womens Health.

[190] Dîră L.-M., Drăguşin R.C., Văduva C.-C., Zorilă G.L., Nagy R.D., Ciobanu Ş.-G., Berbecaru E.-I.-A., Enache I.-A., Iliescu G.D. (2023). Importanţa Endometritei Cronice Şi a Disbiozei În Eşecul de Implantare În Ciclurile de FIV. Obstet. Ginecol..

[191] Kitaya K., Ishikawa T. (2022). Lincomycin Administration against Persistent Multi-Drug Resistant Chronic Endometritis in Infertile Women with a History of Repeated Implantation Failure. Appl. Microbiol..

[192] Khan K.N., De Ziegler D., Guo S.-W. (2024). Bacterial Infection in Endometriosis: A Silver-Lining for the Development of New Non-Hormonal Therapy?. Hum. Reprod..

[193] Brichant G., Laraki I., Henry L., Munaut C., Nisolle M. (2021). New Therapeutics in Endometriosis: A Review of Hormonal, Non-Hormonal, and Non-Coding RNA Treatments. Int. J. Mol. Sci..

